# Unsupervised Descriptive Phenotyping of Cardiometabolic Laboratory Patterns from a Routine Five-Analyte Panel: Stability, Transportability and a Rule-Based Laboratory Interpretation Layer

**DOI:** 10.3390/diagnostics16183025

**Published:** 2026-09-18

**Authors:** Yerlan Suleimenov, Ayat Assemov, Bakhytzhan Seksenbayev, Akezhan Koishybay, Albina Omarova, Baurzhan Nurlan

**Affiliations:** 1 CDL Olymp LLP (Laboratory Network), Head Office, Astana 010000, Kazakhstan; yerlan.suleimenov@kdlolymp.kz (Y.S.); a.koyshybay@kdlolymp.kz (A.K.);; 2 School of Medicine, Nazarbayev University, Astana 010000, Kazakhstan; 3 KazDream LLP, Astana 010000, Kazakhstan

**Keywords:** unsupervised learning, cluster validation, bootstrap stability, model transportability, laboratory medicine, reference intervals, interpretable machine learning, dyslipidemia, dysglycemia, Kazakhstan

## Abstract

(1) **Background:** Reference-interval flags read a lipid–glucose panel one analyte at a time. We asked whether a routine five-analyte panel supports reproducible, transportable, auditable descriptive grouping of laboratory profiles. (2) **Methods**: Retrospective analysis of 21,832 laboratory-order records (adults; glucose, total cholesterol, triglycerides, HDL-C, LDL-C; collection dates not exported) from purchasers of a screening panel in Kazakhstan: log transform, robust scaling, PCA, K-means; eight cluster-number criteria; bootstrap recovery with and without pipeline refitting; frozen-classifier transfer to an identifier-disjoint cohort (n=11,382) with de novo re-derivation; a fully specified reference-interval rule layer. No clinical endpoints were available. (3) **Results**: Four descriptive laboratory-pattern clusters (lower glucose and lipids; higher LDL-C/TC; higher triglycerides, lower HDL-C; marked hyperglycaemia) had bootstrap Jaccard 0.965–0.981 with and without pipeline refitting and were re-derived independently in the validation cohort (ARI 0.82); a five-cluster configuration was not (0.33). Prevalence-shift point estimates were within 5 percentage points (post hoc exploratory benchmark; the C1 interval slightly crossed it). Rule-based reconciliation changed the delivered category of 8.2% of records from their K-means label; flags alone reproduced 77% of reports but 30% of the triglyceride–HDL-C pattern. (4) **Conclusions**: The panel supports a stable descriptive partition; prognostic and clinical validity remain to be shown.

## 1. Introduction

Cardiovascular disease remains the leading cause of death worldwide, and a large share of that burden is attributable to modifiable metabolic risk factors, in particular dysglycaemia and atherogenic dyslipidaemia [1,2]. Routine screening encounters increasingly include a compact biochemical panel—glucose together with the standard lipid profile—that is inexpensive, widely available and already embedded in laboratory information systems. In current practice, these analytes are either collapsed into a single composite risk estimate, using equations such as SCORE2 [3] or the pooled cohort equations [4] that also require blood pressure, smoking status and age, or each compared in isolation, against a fixed reference interval [5].

Both readings discard information the panel contains. A composite score is, by construction, agnostic to the patternof abnormality; analyte-by-analyte flagging ignores the covariance among lipid fractions and glucose that gives recognised laboratory patterns—such as the raised-triglyceride, low-HDL-C profile associated with insulin resistance—their identity [6,7]. Data-driven grouping of patients by the multivariate shape of their results has been used in cardiology to propose subtypes [8,9,10]. Two barriers limit its translation. First, unsupervised clustering is frequently reported without evidence that the partition is reproducible under resampling and across cohorts, so that derived groups may be artefacts of a single fit [11]. Second, an integer cluster label is not, by itself, a laboratory result; the rules that turn it into a report are rarely written down completely. A further, often overlooked, issue is the inclusion of algebraically derived quantities such as the atherogenic index alongside their constituents, which double-counts information [12].

Throughout this article, a *phenotype* is a *laboratory-derived descriptive phenotype*: a recurring pattern of five analyte values, named after the analytes that distinguish it. It is not a diagnosis, a prognostic category or a treatment indication, and nothing in this work establishes construct validity against biology, prognostic validity against outcomes or clinical utility. The analysis contains no diagnoses, medication data, blood pressure, body-mass index, smoking status, HbA1c, renal function or follow-up.

Within that framing we set out to (i) derive descriptive laboratory-pattern clusters from the five primary analytes of a routine panel, excluding derived ratios; (ii) determine how many clusters the data support, separating the statistical question of stable structure from the operational question of how many categories a laboratory report should carry; (iii) assess reproducibility under resampling, transportability of the frozen model to an identifier-disjoint cohort of the same network, and independent re-derivation there; (iv) specify completely a deterministic laboratory interpretation layer that combines cluster membership with age- and sex-specific reference-interval flags, quantifying what the clustering adds over the flags alone. The data come from a laboratory network in Kazakhstan, a setting rarely represented in this literature; we make no claim of national representativeness.

## 2. Materials and Methods

### 2.1. Study Population and Data

This was a retrospective, non-interventional analysis of de-identified, routinely collected results of the “Cardiorisk” biochemical screening panel of CDL Olymp, a clinical laboratory network that centralises testing in 22 laboratories across 20 regions of Kazakhstan and collects specimens at more than 800 collection points. Each record carried a laboratory order number, sex, age, the five panel analytes—glucose, total cholesterol (TC), triglycerides (TG), high-density lipoprotein cholesterol (HDL-C) and low-density-lipoprotein cholesterol (LDL-C), all in mmol/L—and the panel configuration purchased. The order number identifies one order, i.e., one specimen-collection episode; the order record in the laboratory information system also holds the patient’s details, the test list and the time and place of collection and processing, none of which were exported. The panel is sold in three configurations (standard, full or extended); the two larger ones add apolipoproteins B and A1, lipoprotein(a), high-sensitivity C-reactive protein and troponin I, which took no part in clustering and are used in Section 3.10 only. Records came from three exports: two regional centres (Almaty, n=5796; Astana, n=4920) and a network-wide extract (n=17,408) that also contained every Almaty record and was deduplicated against it by order number before any analysis. The two leading digits of the order number encode the collection point (57 points in the analysed cohort). The exports carry no collection dates, instrument, reagent-lot or calibration metadata, so the study period cannot be stated; they also carry no information on repeat measurements, inpatient or outpatient status, acute illness, medication, referral source, urban or rural residence or socioeconomic context, and each record is one specimen-collection episode. Patients are instructed to attend in the morning after an 8–12-h fast; fasting status is not recorded, and the analyte is therefore referred to simply as glucose.

The panel is a purchasable product, and 84.3% of the analysed records carry the promotional variant of its name (84.6% of the regional-centre records; 84.1% of the network-unique records). The cohort is thus a self-selected sample of purchasers of a discounted cardiometabolic panel in six product configurations, not a sample of any defined population, and we describe it as a large laboratory-network sample from Kazakhstan. The order number identifies an order, not a person; the exports contain no person identifier, so repeat orders by the same individual cannot be excluded. Throughout this article, “record” and “profile” denote one laboratory order, and “patient” is the person whose specimen an order contains, without implying that different orders are different persons. As an upper bound, 5.6% of analysed records share sex, age to the day and collection point with another record (Supplementary Table S18).

Age was stored in days and converted to years (division by 365.25 when the recorded value exceeded 200). Sex was harmonised from numeric and textual encodings to the two values the source system offers, male and female, which were recorded for every record; the system has no unknown, intersex or non-binary category, a constraint we note as a limitation. Sex and age were used only for reference-interval selection, never for clustering. Where an analyte arrived under synonymous field names in the concatenated network extract (total cholesterol under two names in 14,043 and 3364 records; the atherogenic coefficient, apolipoproteins and C-reactive protein likewise), the fields were folded into one (Supplementary Table S20 presents the data dictionary).

The provenance of LDL-C is relevant because a calculated LDL-C would be a deterministic function of three other features. In these exports, it is not: regressing LDL-C on TC, HDL-C and TG yields $R^{2}=0.909$, whereas every calculated variant—Friedewald [13], Martin–Hopkins [14] and Sampson [15]—reproduces $R^{2}\geq 0.999$. The mass-balance residual TC−LDL-C−HDL-C−TG/2.2 is wide (SD 0.37 mmol/L; 2.8% of records within 0.01 mmol/L of zero), and LDL-C is reported for all 443 analysed records with TG > 4.5 mmol/L, where calculated values are conventionally suppressed (Supplementary Table S8). This excludes a deterministic calculation and is consistent with the laboratory’s documentation of a directly measured LDL-C. The network measures five analytes on Roche cobas clinical-chemistry modules of two generations operated side by side: c 311, c 501/c 502 and c 702 modules of the cobas 6000/8000 series, and c 503/c 703 modules of the cobas pro series. The newer modules were introduced about three to four years before this study without retiring the earlier ones, and the reagent pack appropriate to each module is used. LDL-C is measured with the homogeneous enzymatic colourimetric third-generation assay (Roche LDL-Cholesterol Gen.3, LDLC3), not calculated. All modules are calibrated with the same lipid calibrator (Roche C.f.a.s. Lipids) and monitored with the same control material (Roche PreciControl ClinChem Multi); the manufacturer’s stated position is that both module generations return equivalent results. The laboratory does not keep a chronology of reagent lots, and the exports do not record the performing laboratory or module, so lot, module and calibration history cannot be reconstructed for individual records. External quality-assessment records for the export period were not available to us. The residual differs between exports (SD 0.31 and 0.29 mmol/L for the two regional centres against 0.41 for the network extract) and between collection points (Section 3.11). With a directly measured LDL-C, a non-zero residual is expected. Its variation between sites is compatible with analytical or preanalytical heterogeneity, but the available data cannot attribute it to a specific cause—module generation, reagent lot, calibration history, specimen handling or case mix. The applicable national legislation and the consent arrangements are set out in the Institutional Review Board and Informed Consent statements.

### 2.2. Cohort Selection and Data Quality

**Figure 1:**
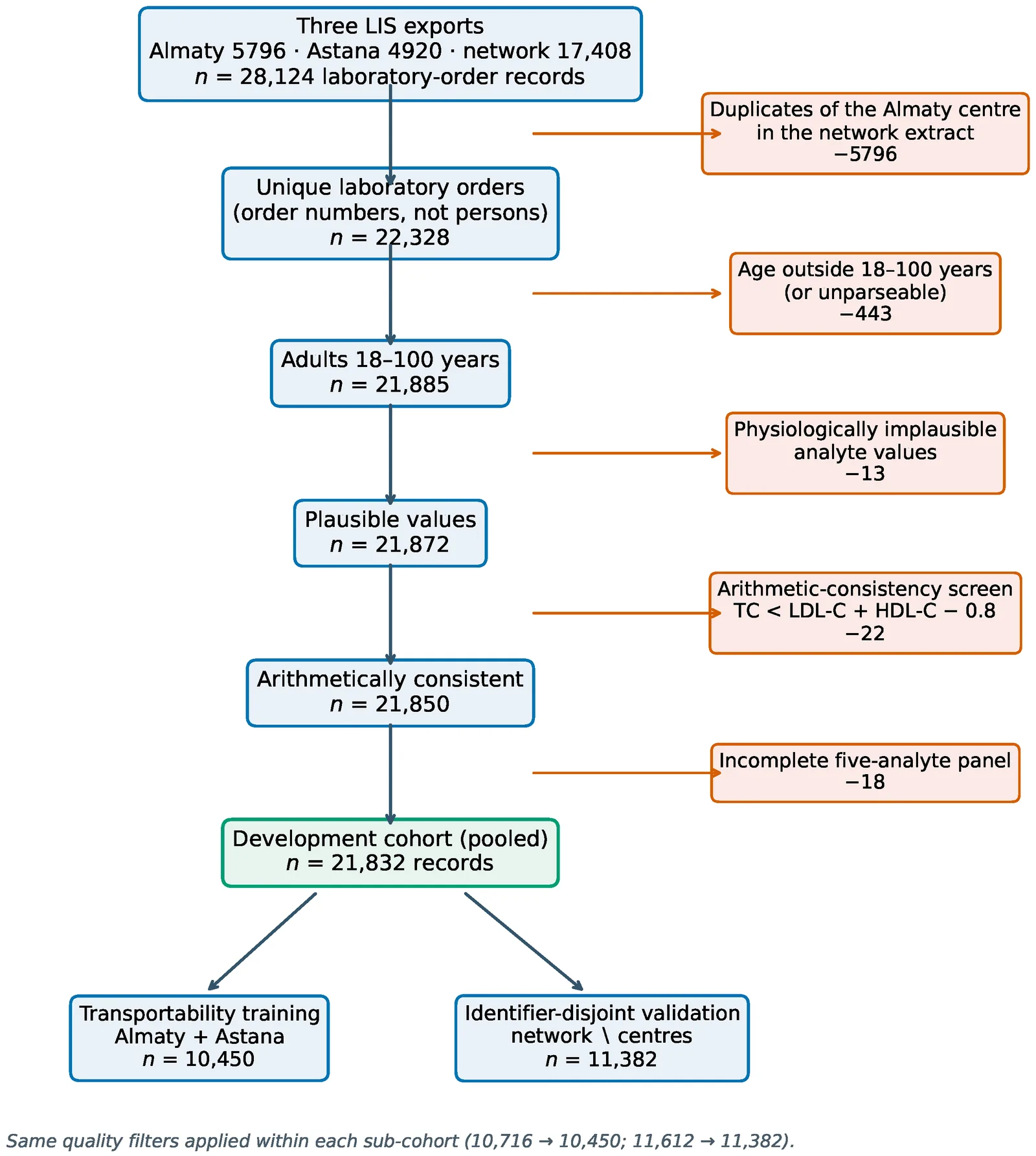
Cohort selection flow. Exclusion steps are applied sequentially to the deduplicated pool; the transportability analysis (Section 3.6) applies the same filters within the two sub-cohorts.

From the deduplicated pool, we retained adults aged 18–100 years and excluded records with physiologically implausible values (glucose outside 1.0–40, TC 1.0–20, TG 0.1–30, HDL-C 0.2–5.0, LDL-C 0.3–15 mmol/L; 13 records), records failing an arithmetic-consistency screen and records with an incomplete five-analyte panel (Figure 1). The screen removes records in which TC<LDL-C+HDL-C−0.8 mmol/L. The tolerance of 0.8 mmol/L was set ad hoc when the panel was deployed, before this study, and we have no principled derivation for it; physically, the excess of LDL-C plus HDL-C over total cholesterol is impossible at any positive value, and zero tolerance would remove 866 further records (4.0%). Section 3.7 and Supplementary Table S13 report the screen at tolerances of 0, 0.2, 0.5 and 0.8 mmol/L and show that the excess occurs in every export, so the deployed screen removes the most extreme physically impossible profiles and no more. Whether the excluded records are enriched for particular laboratory patterns cannot be assessed within the analysed cohort, because they are removed from it; at zero tolerance, the 866 retained records with a smaller excess are distributed across all four clusters. No values were imputed; all exclusion thresholds are documented constants of the analysis code.

### 2.3. Preprocessing

All five features are right-skewed and were transformed with log(1+x), then scaled with a robust scaler (centring on the median, scaling by the interquartile range) [16]. Robust scaling is a modelling choice with consequences, not a neutral default: it provides observations outside the interquartile range—most notably the diabetic glucose range—greater influence than per-feature variance standardisation would, and glucose accordingly carries 40.7% of the scaled variance against 20.0% under variance standardisation (Section 3.4). We report alternative scalings and unscaled and untransformed variants as sensitivity analyses (Section 3.7), and we do not claim that any of them are clinically the correct weighting.

The atherogenic coefficient routinely reported by the laboratory, AC=(TC−HDL-C)/HDL-C [17], was excluded from clustering because it is an exact algebraic function of two features already present; it was retained for the interpretation layer (Section 2.7). The effect of adding it, or the atherogenic index of plasma $\mathrm{AIP}=\log_{10}\left(\mathrm{TG}/\mathrm{HDL}\text{-}C\right)$ [12], is reported in Section 3.7.

### 2.4. Dimensionality Reduction

Principal component analysis (PCA) [18] was applied to the scaled features, and the smallest number of components explaining at least 90% of the variance was retained. PCA was used before K-means for two operational reasons—it decorrelates the three lipid fractions, and it yields a three-axis space in which centroid displacements and the per-patient assignment margin (Section 2.6) can be reported on a common scale—and not because it is expected to improve cluster separation. Because PCA preserves variance rather than cluster structure, the primary partition was compared with K-means run directly in the five-dimensional scaled space (Section 3.7). The transform stack is fitted once on the development cohort and frozen; the resampling analyses state explicitly whether it was refitted.

### 2.5. Clustering and the Number of Clusters

K-means [19] (scikit-learn [16], n_init =20, seed 42) was the primary algorithm and was benchmarked against Gaussian mixture models (GMM; four covariance structures) [20] and HDBSCAN [21]. Eight criteria impact the number of clusters *k*, and we keep two questions apart that they are often answered at once: that is, *k* gives the most reproducible partition (a statistical question), and how many categories a laboratory report should carry (an operational one). Three internal indices were computed over k = 2–8: the silhouette coefficient [22], the Calinski–Harabasz index [23] and the Davies–Bouldin index [24]. The gap statistic against a uniform reference [25] (20 reference draws per *k*) was evaluated over k = 1–7, so that the no-clustering null is inside the grid. Out-of-sample prediction strength [26] was computed over 15 random split-halves per *k* and summarised as the minimum over clusters. The remaining criteria were the Calinski–Harabasz index of the validation cohort over k = 3–5, bootstrap cluster-wise stability [27] at the two candidate values k = 4 and k = 5 and the Bayesian information criterion of the Gaussian mixtures over k = 2–8 for three covariance structures. Unimodality of each retained component was tested with Hartigan’s dip test [28].

On the basis of these criteria (Section 3.3), the primary model has four clusters. A five-cluster configuration, which separates an intermediate-hyperglycaemia group from the four, is reported as a secondary reporting configuration in the Supplementary Material (Table S4) and is used in Section 3.3 to measure, on delivered reports, what the fifth category would and would not change. K-means numbers its clusters arbitrarily, so the fitted solution is anchored by a deterministic relabelling rule applied to the back-projected centroids. The cluster with the highest glucose centroid becomes C3 (marked hyperglycaemia). Of the remaining three, the one with the lowest total cholesterol becomes C0 (lower glucose and lipids); of the last two, the one with the higher HDL-C becomes C1 (higher LDL-C/TC) and the remaining one C2 (higher triglycerides, lower HDL-C). In the five-cluster configuration, the second-highest glucose centroid becomes F4 (intermediate hyperglycaemia) and F0–F3 are named by the same rule, so that C0–C3 and F0–F3 denote corresponding patterns. The rule makes the integer-to-label mapping, and every interpretation rule that depends on it, invariant to retraining.

### 2.6. Stability, Uncertainty, Transportability and De Novo Re-Derivation

Numerical reproducibility was quantified by refitting K-means under eight seeds distinct from the reference and computing the adjusted Rand index (ARI) against the reference partition [11]; with 20 restarts, this measures optimiser determinism, not validity, and is reported alongside the resampling measures. Bootstrap cluster-wise stability followed Hennig [27]: 100 resamples of size *n* drawn with replacement at the record level (duplicated records kept as duplicates in fitting and evaluation), K-means refitted on each and every reference cluster matched to the resample cluster with the largest Jaccard index (best match, without a one-to-one constraint). Best matching is Hennig’s cluster-wise definition: it asks, for each reference cluster separately, whether some resample cluster recovers it. A one-to-one (Hungarian) constraint would force a reference cluster to accept a poorer partner whenever its best partner is claimed by another cluster, and would lower the reported Jaccard of the less stable cluster without changing whether it was recovered. The two procedures can differ only when a cluster is recovered at Jaccard below 0.5, because two disjoint reference clusters cannot both share more than half of their union with the same resample cluster. For the four-cluster solution, this never occurred (Section 3.5), so its best-match and one-to-one results coincide; the difference is confined to the dissolving fifth cluster of the secondary configuration. We report means, medians, 10th percentiles, 95% intervals and the share of resamples in which a cluster is recovered at Jaccard below 0.5 (“dissolved”). The procedure was run in two forms: on the frozen projection (scaler and PCA of the full cohort), and with the whole log–robust-scaler–PCA stack refitted inside every resample, so that the pipeline, rather than only the partition, is resampled.

Assignment uncertainty was quantified for every patient by the relative margin $\left(d_{2}-d_{1}\right)/d_{1}$, where $d_{1}$ and $d_{2}$ are the distances to the nearest and second-nearest centroid in the PCA space, and by a border flag (distance to the assigned centroid exceeding 1.5 times that cluster’s median assignment distance). The 1.5 multiplier was chosen when the interpretation layer was designed, by inspection of the assignment-distance distributions and before any result of the layer was examined; it was not prespecified in a protocol, and its sensitivity over 1.25–2.0 is reported.

Transportability was assessed as leakage-free: the pipeline was fitted on the two regional centres only (n=10,450), frozen and applied to the identifier-disjoint remainder of the network extract (n=11,382). Validation centroids were matched to training centroids by the Hungarian algorithm; prevalence differences are reported in percentage points with Newcombe 95% intervals built from Wilson intervals and centroid displacements in units of the training within-cluster standard deviation per axis. Because a statistically significant shift is not necessarily an operationally important one, we set two operational benchmarks: a prevalence shift of at most 5 percentage points per cluster and a centroid displacement of at most 0.5 within-cluster SD on every axis. They were set after the first version of this analysis and before the present verdicts were read; they are post hoc exploratory operational benchmarks, not prespecified validation criteria, and are referred to as such throughout. No benchmark was set for the per-feature distribution statistics (Wasserstein and Kolmogorov–Smirnov), which are reported descriptively. Because projection onto fixed centroids cannot fail to populate the pre-existing clusters, the model was additionally re-derived de novo in the validation cohort for both k=4 and k=5. The de novo clusters were matched to the development centroids on standardised profiles and compared with the projected solution by ARI and matched agreement. Two de novo models on random halves of the development cohort give the no-population-shift reference.

### 2.7. Rule-Based Laboratory Interpretation Layer

Cluster assignment is embedded in a deterministic report generator that we describe as *laboratory* interpretation: it comments on laboratory values and patterns and does not integrate symptoms, history, medication or examination. Each analyte is flagged HIGH, LOW or NORMAL against age- and sex-specific reference intervals (Appendix A). These are the population reference intervals that were in use in the network’s reporting system before this study; their provenance is visible in their values (44 of the 50 limits are round numbers in mg/dL), and they are *not* guideline decision limits or treatment targets—a distinction the reports state. Auditing the table exposed two defects in its glucose row: a non-monotone upper limit of 6.38 mmol/L for ages 61–70, above the 6.10 of the older band and above the impaired-fasting-glucose threshold, and a lower limit rising with age. We corrected both. All primary results use the corrected table; the deployed table is retained for the sensitivity analysis of Section 3.9. Thirteen single-deviation templates (Appendix B), eight combination rules that select narrative fragments without changing the category (Appendix C), and an arithmetic-consistency check complete the layer. Six de-identified patient-level examples are traced through every step of the layer in Supplementary Table S21.

A two-stage reconciliation harmonises the statistical label with the flags. Stage 1 applies to border cases only; stage 2 applies to every patient and is iterated to a fixed point (at most three passes). The rules are data, not code. The complete decision table—every rule with its identifier, source category, condition, target and the number of records it moved—is printed in Supplementary Table S11a, and the whole layer is specified as pseudocode in Supplementary Section S23. Every delivered report can therefore be reproduced from five values, sex and age, without the source code. The layer uses one guideline decision limit: a rule that moves a patient into the marked-hyperglycaemia category fires only when the glucose flag is HIGH, *and* the value is at or above 7.0 mmol/L, the fasting-plasma-glucose threshold for diabetes [29,30]. This limit was introduced after we found that the flag-only rules of the original submission moved 697 patients with a median glucose of 6.39 mmol/L into that category (Section 3.8). A patient whose glucose is flagged HIGH but lies below 7.0 keeps the lipid-pattern category and receives the glucose template, or the glucose flag alongside the category passport. The rules were written by the laboratory’s clinical staff on the development data and evaluated on the same data; they were not prespecified, and their behaviour on the identifier-disjoint cohort under the frozen model is reported as the only available external check (Supplementary Table S11a).

To quantify what the clustering contributes, a flags-only baseline was computed: every patient is assigned by the reconciliation rules alone, starting from C0 with no cluster input, and agreement with the full system is reported overall and per category.

### 2.8. Implementation and Reproducibility

All analyses used Python 3.11 with NumPy 2.4.4, pandas 3.0.2, scikit-learn 1.8.0 [16], SciPy 1.17.1, Matplotlib 3.10.9, Seaborn 0.13.2 and joblib 1.5.3 (exact versions pinned in the deposit’s requirements.txt); seeds were fixed throughout. The serialised scaler, PCA and K-means objects are reused unchanged at inference; the relabeling map, the per-cluster median assignment distances and the decision table are serialised with the model. The interpretation layer is covered by 31 unit tests. One script regenerates the tables and figures of the main analysis from the raw exports, and six further scripts produce the additional analyses; a verification script checks every number quoted in this article and its Supplementary Material against the machine-readable outputs. The software is proprietary to the laboratory network and is deposited as a restricted-access record (Data Availability); the parameters needed to re-implement the frozen model without it—scaler centre and scale, PCA components and mean, centroids, reference intervals and decision table—are reported in full. Reporting follows STROBE [31] (checklist in the Supplementary Material). TRIPOD+AI [32] is not applicable: this work develops no prognostic model and has no discrimination or calibration to report. Use of generative artificial intelligence is disclosed in the Acknowledgements.

## 3. Results

### 3.1. Cohort Characteristics

The three exports comprised 28,124 records, of which 5796 were duplicates of the Almaty centre contained in the network-wide extract, leaving 22,328 unique orders. The filters excluded 443 records with age missing or outside 18–100 years, 13 with implausible values, 22 failing the arithmetic screen and 18 with an incomplete panel, so that 21,832 laboratory-order records of adults, one five-analyte profile each, entered the analysis (Figure 1; Supplementary Table S1). Median age was 53.3 years (interquartile range 41.6–64.0) and 59.9% were women. Analyte distributions are summarised in Table 1; mean total cholesterol (5.36 mmol/L) and LDL-C (3.40 mmol/L) lay above the guideline goal values for adults at low risk [7], as expected of purchasers of a cardiometabolic panel.

### 3.2. Latent Structure

Three principal components explained 92.8% of the variance (45.4%, 29.2% and 18.2%). PC1 was a glycaemic axis dominated by glucose (loading 0.90); PC2 was a cholesterol axis loaded on TC (0.68) and LDL-C (0.66); PC3 was the triglyceride–HDL-C balance (TG +0.66, HDL-C −0.64; Supplementary Table S2). All three components are unimodal by Hartigan’s dip test (p≥0.99; Supplementary Table S7): in the space in which the clustering is performed, the data form a continuum rather than separated groups. Three of the five analyte marginals, taken singly, reject unimodality—HDL-C ($p=2.6\times 10^{-5}$), triglycerides (p=0.002) and glucose (p=0.010)—as a mixed-sex screening population with a prediabetic shoulder would produce.

### 3.3. The Number of Clusters

**Figure 2:**
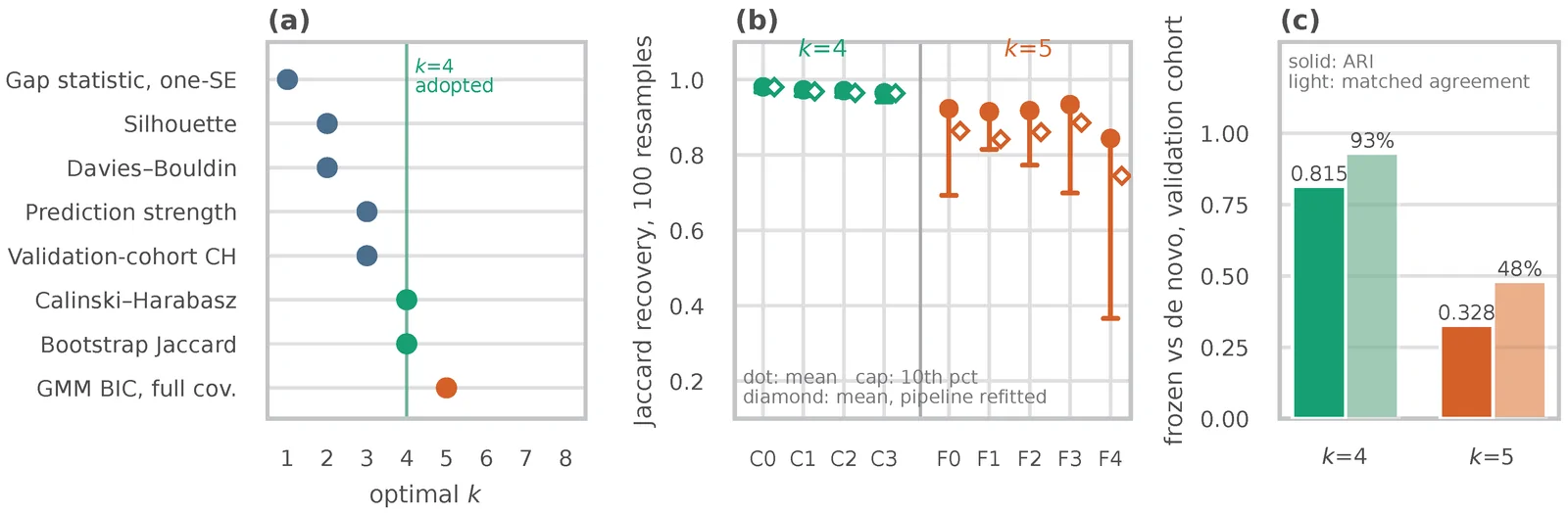
The number of clusters. (**a**) The value of *k* at which each of the eight criteria is optimal (blue, a value below four (one, two or three); green, four; orange, five). The grids differ by criterion: gap statistic over k=1–7, internal indices and mixture BIC over k=2–8, prediction strength over k=2–6, the validation cohort’s Calinski–Harabasz index over k=3–5 and the bootstrap at the two candidate values only. (**b**) Best-match Jaccard recovery of each cluster over 100 bootstrap resamples: dot, mean; cap, 10th percentile; hollow diamond, mean when the whole log–robust-scaler–PCA pipeline is refitted inside every resample. C0–C3, primary model; F0–F4, five-cluster configuration. (**c**) Agreement between the frozen centres-only model and a model re-derived de novo in the identifier-disjoint validation cohort: adjusted Rand index (solid) and matched agreement (light), for k=4 and k=5.

The eight criteria and what each selects are shown in Figure 2a and Table 2 (Supplementary Tables S5, S6 and S10c). The silhouette and the Davies–Bouldin index are optimal at k=2 (0.499 and 1.003). The Calinski–Harabasz index of the pooled cohort peaks at k=4 (9293; 9.2% above its value at k=5). The gap statistic under the one-standard-error rule, evaluated over k=1–7, selects k=1 (gap 2.301 against 2.198 at k=4); restricted to k≥2, it selects k=4. Prediction strength over 15 splits is highest at k=3 (0.913 ± 0.021) and not distinguishable from k=4 (0.898 ± 0.014); it falls to 0.776 ± 0.056 at k=5. On paired splits, the k=5 minus k=4 difference is −0.122±0.052 (t=−2.34, 14 d.f.). The validation cohort’s Calinski–Harabasz index is nominally highest at k=3 (5015 against 5005 at k=4 and 4577 at k=5). Bootstrap recovery is uniformly higher at k=4 than at k=5 (Section 3.5). The one criterion that selects five is the BIC of a full-covariance Gaussian mixture (179,677 at k=5); its diagonal and tied variants select k=8 and k=7.

The optima are therefore 1, 2, 2, 3, 3, 4, 4 and 5: no number of clusters is identified, which is the signature of a continuum, and on a continuum, the number of categories in a report is a design parameter rather than an estimate. Among the values considered for reporting, every criterion except the mixture BIC ranks four above five. We therefore adopt four clusters as the primary model on the statistical question and treat the five-cluster configuration—which separates an intermediate-hyperglycaemia group F4 (n=2454, 11.2%) from the four—as a candidate answer to the operational question, to be judged on the reports it delivers. The two partitions differ in the raw label for 14.8% of the cohort (ARI 0.767); F4 is drawn from C2 (54.0%), C0 (24.9%), C3 (17.2%) and C1 (3.9%) (Supplementary Table S4).

The operational case for the fifth category is that, without it, 610 patients with an intermediate glucose (those absorbed into C0 at k=4) might receive a reassuring report. Measured on the delivered reports rather than the raw labels, this does not happen. Under the corrected reference table, 523 of the 610 receive the glucose single-deviation template at k=4, and 510 receive the intermediate-hyperglycaemia passport at k=5. Thirty-four receive the C0 passport under both configurations, and none of those has a glucose of 6.1 mmol/L or above. The number of patients issued a C0 passport without a glucose flag at a glucose of 6.1 mmol/L or above is zero under both configurations with the corrected table. It is 28 under both with the deployed table—all aged 61–70 with glucose 6.11–6.37 mmol/L, the defect of Section 3.9. The choice of *k* therefore does not change whether an intermediate glucose is reported; it changes whether it is reported as a category or as a flag. What the fifth category costs is measured in Section 3.5 and Section 3.6: it is the least reproducible cluster under resampling, and it is not re-derived in the validation cohort. On that evidence, the operational question is answered the same way as the statistical one, and the delivered report of the primary system carries four categories.

Gaussian mixtures reached comparable silhouettes (0.23–0.26 across covariance structures) but assigned 39–58% of patients with a maximum posterior probability below 0.7, and HDBSCAN labelled 69–100% of patients as noise at every parameterisation (min_cluster_size 50–500)—further evidence of a continuum without dense cores. The low posterior confidence of the mixtures is an honest estimate of assignment ambiguity that a hard label conceals; rather than adopting soft membership, we report that ambiguity per patient through the margin and the border flag (Section 3.5).

### 3.4. The Four Clusters

**Figure 3:**
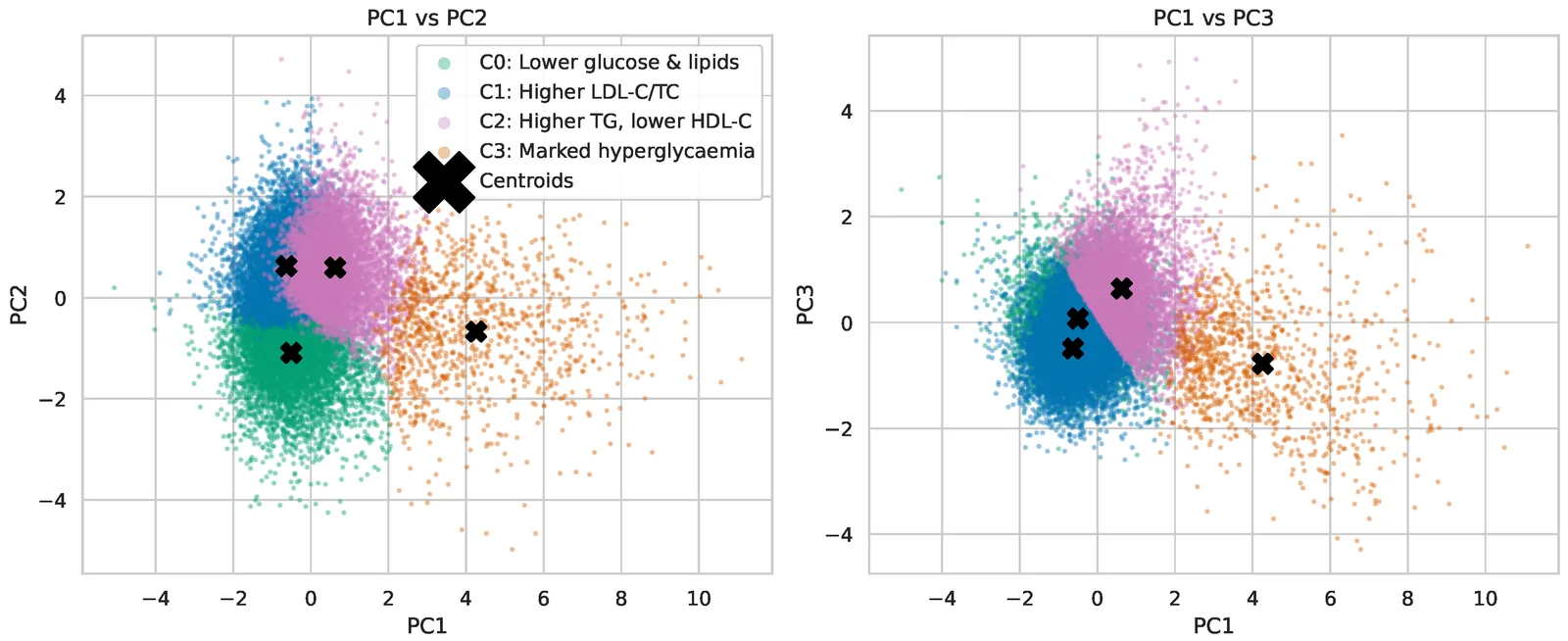
The four clusters in the PCA space: (**left**) PC1 vs. PC2 and (**right**) PC1 vs. PC3: Black crosses denote centroids. Axis scales are retained because centroid displacements are quoted in these units. Clusters are separated along the glycaemic (PC1), cholesterol (PC2) and triglyceride–HDL-C (PC3) axes with smooth rather than gap-separated boundaries. Cluster labels name single-measurement laboratory patterns, not diagnoses.

**Figure 4:**
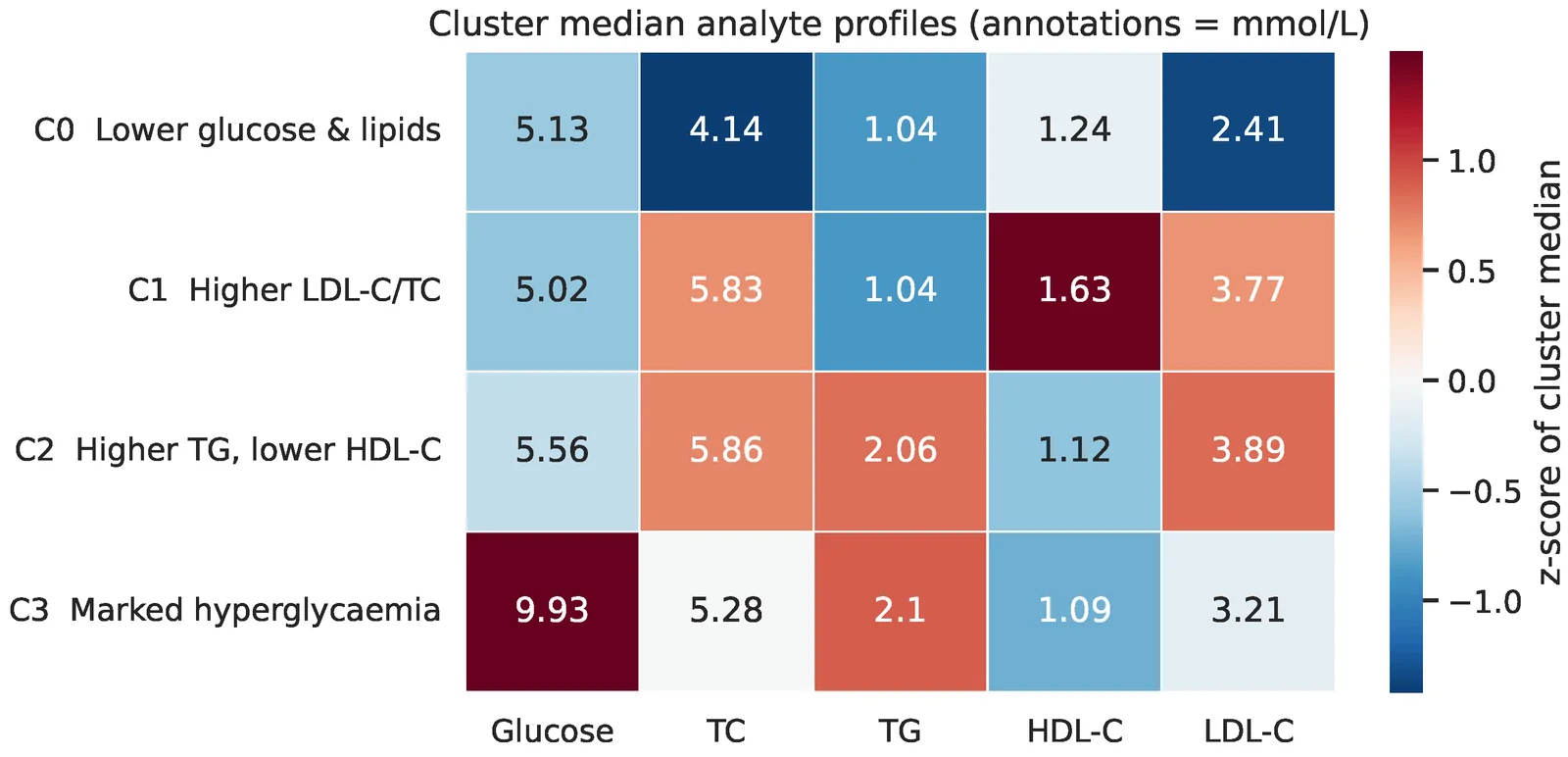
Standardised (z-scored) cluster-median analyte profiles; cell annotations give the original median values (mmol/L). Each cluster is defined by a distinct combination of glycaemic and lipid deviations. Cluster labels name single-measurement laboratory patterns, not diagnoses.

**Figure 5:**
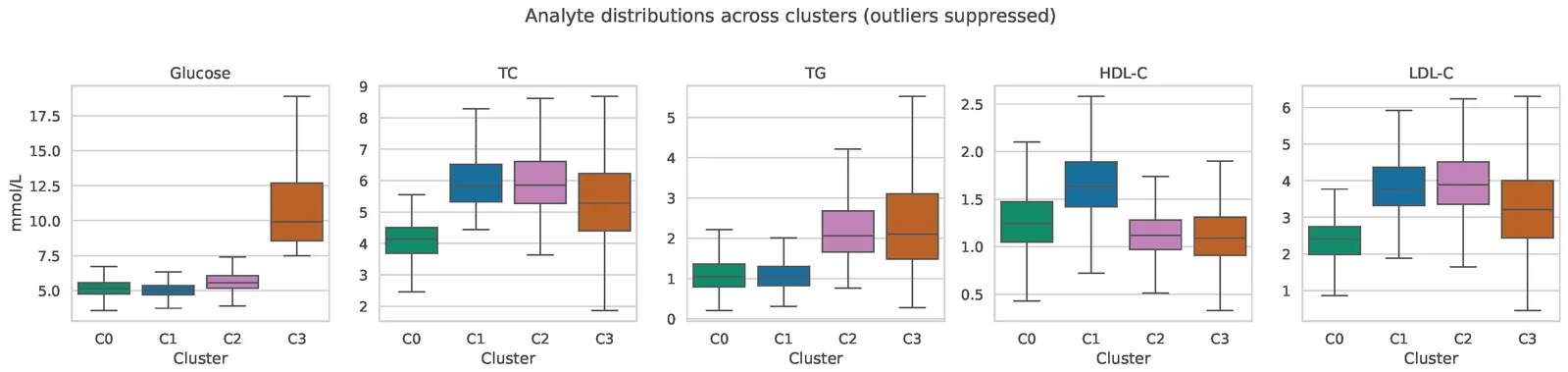
Distribution of each analyte across the four clusters. Boxes provide the interquartile range with the median; whiskers extend to 1.5 times the interquartile range and observations beyond them are not drawn—a suppression that is largest for glucose in C3. The clusters differ on different analytes, i.e., they encode multivariate patterns rather than a single severity axis. Cluster labels name single-measurement laboratory patterns, not diagnoses.

The four clusters and their median analyte profiles are provided in Table 3 and Figure 3, Figure 4 and Figure 5. Their labels describe laboratory patterns and carry no risk, diagnostic or treatment claim.

- **C0—Lower-glucose, lower-lipid profile (32.3%):** the lowest TC and LDL-C, TG as low as in C1 and glucose in the lower range; the reference pattern against which single deviations are reported.
- **C1—Higher LDL-C/total-cholesterol profile (34.5%):** elevated TC and LDL-C with triglycerides in the lower range and the highest HDL-C; 75.0% women.
- **C2—Higher-triglyceride, lower-HDL-C profile (28.5%):** raised TG with the lowest HDL-C, raised LDL-C/TC and glucose in the upper normal range; the pattern usually described as atherogenic dyslipidaemia, here named by its analytes.
- **C3—Marked hyperglycaemia with adverse lipid profile (4.7%):** median glucose 9.93 mmol/L with raised TG and low HDL-C; the oldest group (median 60.4 years). A single, nominally fasting glucose in this range is a laboratory pattern, not a diagnosis, which requires confirmatory testing [30].

The clusters are not supported equally by the five analytes. Glucose carries 40.7% of the scaled variance and 47.7% of the between-cluster separation, almost all of it through C3; with C3 excluded, its share falls to 7.3%, and the remaining three clusters are separated by total cholesterol (28.2%), LDL-C (25.0%), triglycerides (23.9%) and HDL-C (15.6%) (Supplementary Table S3). The model is therefore most accurately read as a three-way lipid partition plus a small hyperglycaemia-defined group. This weighting follows from the robust scaler: under variance standardisation glucose carries 20.0% of the scaled variance and 8.2% of patients change cluster (ARI 0.786; Section 3.7).

Age and sex differ across clusters (Supplementary Table S12). Women are 59.9% of the cohort but 75.0% (95% CI 74.1–76.0) of C1 and 47.8% (46.5–49.0) of C2; we did not investigate why and offer no explanation. Within sex and age strata the cluster prevalences differ—C1 is 43.2% of women and 21.4% of men, C2 22.7% and 37.1%—and a K-means model refitted within each stratum on the frozen projection agrees with the primary labels to ARI 0.73 in women, 0.56 in men, 0.63 at ages 18–39, 0.86 at 40–59 and 0.82 at 60 and over. The partition is thus not identical within every subgroup, most visibly in men, where the higher-LDL-C/TC pattern is less common, and the boundary between C1 and C2 is drawn differently. Sex- or age-specific models were not pursued because the reference intervals of the interpretation layer are already sex- and age-specific, and the purpose of a single frozen classifier is to give one patient one answer.

### 3.5. Stability and Assignment Uncertainty

Across eight non-reference seeds, the partition was numerically reproducible (mean ARI 0.988, minimum 0.977); over four of those seeds, the delivered category after reconciliation was equally reproducible (0.987, minimum 0.983). The substantive evidence is resampling-based (Figure 2b; Supplementary Table S6). On the frozen projection, the four clusters were recovered over 100 bootstrap resamples with mean Jaccard indices of 0.965–0.981 (10th percentiles 0.941–0.967; 95% intervals 0.913–0.995; no resample below 0.5 for any cluster). Refitting the whole pipeline inside every resample left them essentially unchanged (0.964–0.980; 10th percentiles 0.941–0.968). The five-cluster configuration was less stable on the frozen projection (F0–F3 0.915–0.934; F4 0.844, 10th percentile 0.366, dissolved in 11% of resamples) and deteriorated when the pipeline was refitted (F0–F3 0.842–0.886; F4 0.745, 10th percentile 0.181, dissolved in 23% of resamples; F1 dissolved in 23%). The four-cluster solution is thus a property of the pipeline, not only of the projection; the fifth cluster is not.

Per patient, the relative assignment margin $\left(d_{2}-d_{1}\right)/d_{1}$ had a median of 0.70 (interquartile range 0.29–1.34); 9.4% of patients had a margin below 0.10 and 22.2% below 0.25, i.e., roughly one patient in five sits close to a boundary, most often with C1 or C2 as the second-nearest cluster (Supplementary Table S16). The border flag marks 16.5% of patients, but only 34% of the patients with a margin below 0.10 carry it: the flag measures distance from the cluster centre, the margin closeness to a neighbour, and the two are complementary. Both are written to the output record, and we regard the margin as the better single expression of the ambiguity that a hard label conceals.

### 3.6. Transportability to an Identifier-Disjoint Cohort and De Novo Re-Derivation

The primary model of Section 3.4, Section 3.5, Section 3.6, Section 3.7 and Section 3.8 is a development-and-description model fitted on the pooled cohort, 47.9% of which comes from the two regional centres. The transportability assessment uses a separate model fitted on the centres alone (ARI 0.901 against the pooled model on the centre records, 96.4% keeping their cluster) and applied frozen to the identifier-disjoint network cohort. The latent geometry transferred. The de novo PCA axes of the validation cohort were congruent with the training axes (absolute cosines 0.995, 0.993, 0.999), and three components again explained 92.7% of the variance. Hungarian matching of validation to training centroids coincided with the identity mapping, and the matched centroids were displaced by at most 0.35 within-cluster SD on any axis (Table 4). Prevalences shifted: C1 by −3.87 percentage points (95% CI −5.11 to −2.63), C2 by +2.87 (+1.65 to +4.09), C3 by +1.88 (+1.29 to +2.46) and C0 by −0.87 (−2.11 to +0.37). All four point estimates lie within the 5-point benchmark and all four displacements within the 0.5-SD benchmark (Supplementary Table S10b). The lower confidence limit for C1 (−5.11) slightly crosses the five-point benchmark: for that cluster, the post hoc exploratory benchmark is met by the point estimate but not by the whole interval. The relative increase of the smallest cluster, C3, is 44%, which is what a 1.9-point absolute shift means for a category of 4%. Per-feature shifts between matched clusters were modest (Wasserstein on the log scale: median 0.017, maximum 0.034; maximum KS 0.15) and concentrated in glucose (mean KS 0.110 against 0.037–0.060 for the lipids; Supplementary Table S14). The precise statement is therefore that the latent axes and the approximate cluster geometry were similar in the two cohorts, while the prevalences of the categories were not identical, most visibly for the two glucose-related clusters.

Re-deriving the model de novo in the validation cohort, the four-cluster solution agreed with the projected frozen classifier at ARI 0.815 and 93.1% matched agreement, and the naming rule identified the same four clusters (centroid matching coincided with the names). The de novo prevalences differed from the training cohort by −1.6%, +1.3%, −2.7% and +20.1% relative for C0–C3. The no-population-shift reference—two de novo models on random halves of the development cohort—is ARI 0.950. The five-cluster configuration did not re-derive: ARI 0.328 and matched agreement 48.1%. Its naming rule failed because the gap between the second- and third-highest glucose centroids is 1.49 mmol/L in the development cohort and 0.19 mmol/L in the validation cohort, so that the cluster the rule would call F4 has the triglyceride–HDL-C pattern (Figure 2c; Supplementary Table S10c). The four-cluster solution is thus independently reproducible across the two cohorts; the five-cluster solution is a property of the development cohort’s local density. Deployment of any such model should nevertheless be as a frozen classifier with prevalence monitoring, since on a continuum re-derived boundaries follow the density they are fitted to.

### 3.7. Sensitivity Analyses

Nine alternative models were compared with the primary solution by Hungarian label alignment (Table 5; Supplementary Table S9). Clustering directly in the five-dimensional scaled space, without PCA, reproduced the partition almost exactly (ARI 0.958; 98.5% of patients keeping their cluster), so the reduction to three components is a convenience, not a determinant of the result. The scaling is decisive: variance standardisation of the log-transformed analytes moved 8.2% of patients (ARI 0.786), the unscaled log transform 30% (0.452) and the untransformed analytes in mmol/L, robustly or variance-scaled, 29–32% (0.364 and 0.373). The choice of scaling is thus the single most consequential preprocessing decision in the pipeline, and we present robust scaling as a stated design choice with these consequences rather than as a clinically justified weighting. Adding the derived atherogenic coefficient moved 8.4% of patients (ARI 0.769), and adding the atherogenic index of plasma moved 6.4% (ARI 0.823), at unchanged separation; because both are exact functions of features already present, this is geometric redistribution rather than new information. Removing LDL-C left the partition largely intact (ARI 0.708; 89.2% agreement). Trimming each analyte at its 0.5th and 99.5th percentiles (847 records, 3.9%) moved 5.0% of the retained patients (ARI 0.880) and shifted the C3 share from 4.7% to 6.0% with a centroid glucose of 9.12 mmol/L; C3 is defined by extreme glucose, so this is expected in direction and modest in size.

### 3.8. The Interpretation Layer

Applied to the development cohort with the corrected reference table, 82.6% of patients carried at least one reference-interval flag. Stage 1 of the reconciliation moved 601 patients (2.8%), stage 2 moved 1210 (5.5%) and 24 were moved by both, so the delivered category differed from the K-means label for 1787 patients (8.2%; Supplementary Tables S11a–b). The delivered distribution was 37.4% C0, 31.9% C1, 25.3% C2 and 5.4% C3 against 32.3/34.5/28.5/4.7% by K-means: the rules move patients with no flag at all from C1 and C2 to C0 (rules R4-06 and R4-08, 1023 and 123 patients) and border patients of C2 without a triglyceride or HDL-C flag to C1 (B4-06, 483). The most frequent single-deviation templates were isolated high total cholesterol (T3, n=2783), isolated low LDL-C (T12, n=2022) and a raised atherogenic coefficient (T6, n=1149); the isolated high-glucose template T2 was issued to 905 patients. The boundary threshold is not decisive: over 1.25–2.0 times the median assignment distance, the delivered category changed for at most 2.0% of patients relative to the primary setting (Supplementary Table S11c). Table 6 summarizes the layer by stage and cohort; the firing count of every rule, the before-and-after category distributions and the training-versus-validation rates under the frozen centres-only model are in Supplementary Tables S11a,b.

The glucose decision limit matters. Under the flag-only rule set of the original submission (any HIGH glucose flag qualified), 697 patients were moved into the marked-hyperglycaemia category by a HIGH glucose flag together with a high triglyceride or LDL-C flag; their median glucose was 6.39 mmol/L, only 21.1% were at or above 7.0 mmol/L and 184 were below 6.1. With the 7.0 mmol/L limit, 147 patients are moved (all at or above 7.0; median 7.34), and the other 550 keep their lipid-pattern category with the glucose flag reported alongside it (Supplementary Table S11d). Under the frozen centres-only model, the rules behaved alike in the two cohorts, changing 7.8% of labels in the training and 8.3% in the validation cohort, with every rule firing at a similar rate per 1000 records (Table 6; Supplementary Table S11a).

The flags-only baseline reproduced 76.9% of delivered categories (ARI 0.542), but asymmetrically: it recovered every C0 assignment, 92.0% of C1, 45.5% of C3 and 30.3% of C2. The mechanism is visible at the cluster medians (Supplementary Table S15): the median C2 patient raises HIGH flags only for total cholesterol and the atherogenic coefficient, because a median LDL-C of 3.89 mmol/L is NORMAL under every LDL-C band of the table and a median TG of 2.06 is NORMAL against a limit of 2.30. Population reference intervals describe a reference population and are wide relative to decision limits: LDL-C is flagged HIGH by the table in 4.1% of the cohort, against 62.4% at or above the 3.0 mmol/L guideline goal for high-risk patients [7], and within C2 in 4.9% against 88.3%; a single guideline rule—TG above 1.7 mmol/L with sex-specific low HDL-C—covers 44.5% of C2, more than the flags-only baseline. Total cholesterol is flagged HIGH in 11,498 patients and LDL-C in 892, all of whom also carry the TC flag; the concordant TC flag is pruned before reporting, and the rules use TC HIGH because LDL-C HIGH alone almost never fires under this table. What the clustering adds over interval-by-interval flagging therefore depends on which intervals the flags use; with the intervals in use, it is the recognition of the triglyceride–HDL-C pattern, whose individual analytes each sit within their reference limits.

### 3.9. The Reference Table: Two Defects and Their Correction

Under the deployed table, the cohort carried 616 LOW glucose flags, of which 422 belonged to patients whose glucose was at or above 3.89 mmol/L (the rising lower limit), and 3852 HIGH flags; the non-monotone upper limit for ages 61–70 left 28 patients with a glucose of 6.11–6.37 mmol/L holding a reassuring C0 passport. Under the corrected table—a lower limit of 3.89 mmol/L for all adults, and an upper limit of 5.83 mmol/L up to age 60 and 6.10 mmol/L above it—there are 194 LOW flags, none non-hypoglycaemic, 4120 HIGH flags and no C0 passport without a glucose flag at a glucose of 6.1 mmol/L or above (Supplementary Table S19). The correction moved the net reconciliation rate from 8.13% to 8.19% of patients and the flags-only agreement by 0.05 percentage points; the clustering does not depend on it, the reports do. At the ADA prediabetes threshold of 5.6 mmol/L [30], 401 patients still receive the C0 passport without a glucose flag because the corrected upper limits (5.83 and 6.10 mmol/L) are population reference limits and not that threshold; whether the laboratory should report against 5.6 is a decision about the table’s purpose (Section 4.6). Three further irregularities are documented in Supplementary Table S19 and left unchanged because they err toward flagging or require a clinical decision. The LDL-C upper limit falls above age 70 (67 HIGH flags in patients over 70 arise only from that fall). The triglyceride limit widens from 2.30 to 3.70 mmol/L above age 66 (400 patients over 66 with a value between the two are not flagged). And eight of twelve sex-by-age groups fall through to the default atherogenic-coefficient limit (Appendix A).

### 3.10. Clusters Against Analytes Not Used in Clustering

The full and extended panel configurations, purchased by 9.7% of the cohort, had analytes that took no part in clustering; because the subset is selected by product choice, the following is hypothesis-generating (Supplementary Table S17). Apolipoprotein B (n=2123) had median values of 0.80, 1.11, 1.25 and 1.17 g/L in C0–C3 (Kruskal–Wallis $p<10^{-200}$) and the ApoB/ApoA1 ratios 0.59, 0.68, 0.93 and 0.80; apolipoprotein A1 was highest in C1 (1.61 g/L), consistent with its HDL-C; high-sensitivity C-reactive protein rose from C1 and C0 through C2 to C3 ($p=5\times 10^{-25}$; unit as exported; the export carries no unit and the laboratory did not confirm one, so only the ordering is interpreted); lipoprotein(a) (n=129) and troponin I (n=336) did not differ. The ordering C0 < C1 < C2 on ApoB and its ratio is the ordering the lipid patterns would predict; it is consistent with the clusters describing something the five analytes did not fully contain, and it is not a validation against outcomes.

### 3.11. Collection-Site Heterogeneity

The analysed cohort comes from 57 collection points, 26 of which have at least 200 records (Supplementary Table S18). Across those 26, the Friedewald residual SD ranged from 0.18 to 1.05 mmol/L and the $R^{2}$ of LDL-C on its fellow analytes from 0.52 to 0.98; the share of records in which LDL-C plus HDL-C exceeds total cholesterol by any amount ranged from 0 to 15.7%. Cluster prevalences also varied (C0 23–42%, C1 15–46%, C2 23–38%, C3 2.8–12.1%; $\chi^{2}=414$ on 75 d.f., Cramér’s V=0.084), the median glucose from 5.02 to 5.65 mmol/L. Since every site is served by the same network, the same reagent system and the same lipid calibrator (Section 2.1), differences of this size point to module-generation, reagent-lot, calibration, specimen-transport or case-mix differences between collection points that these exports cannot separate. Their consequence for the present work is stated in Section 4.8: part of the prevalence shift between cohorts, and of the residual differences between exports, is analytical rather than biological.

## 4. Discussion

### 4.1. Principal Findings

Using the five analytes of a routine lipid–glucose panel, we derived four descriptive laboratory-pattern clusters. They are reproducible under resampling and unchanged when the whole preprocessing pipeline is resampled. They transport as a frozen classifier to an identifier-disjoint cohort of the same network, with prevalence-shift point estimates within post hoc exploratory benchmarks (one confidence interval, C1, slightly crossed the 5-point benchmark). Furthermore—the strongest evidence this design can give—they are re-derived independently in that cohort (ARI 0.82). A fifth cluster, separating intermediate from marked hyperglycaemia, is not reproducible on any of these counts and is reported only as a configuration that a laboratory could choose for its reports. The cluster profiles resemble lipid–glucose patterns described in the dyslipidaemia and metabolic-syndrome literature [6,7]; this resemblance is a description, not a validation. We also specified the rule layer that turns a cluster label into a laboratory report, corrected two defects in its reference table, added the one decision limit it uses, and measured that flags alone reproduce three quarters of its output but miss most of the triglyceride–HDL-C pattern.

### 4.2. What the Clusters Are and Are Not

The clusters are groupings of single-measurement laboratory profiles in a self-selected screening sample. They may be useful for organising a laboratory report and for generating hypotheses; they have not been shown to represent biological subtypes, to predict diabetes or cardiovascular events or to improve any decision. Construct validity, prognostic validity, clinical utility and net benefit have not been demonstrated, and the labels were chosen to make no such claim: C0 is not “optimal” and C3 is not a diagnosis. Two features of the data reinforce this. Glucose and triglycerides are both sensitive to fasting status, recent intake, acute illness and medication [33]; patients are instructed to fast, but this is not verified, and without repeat measurements the clusters describe a profile on one occasion, not a persistent state. And no treatment information was available, so a proportion of C0 and C1 may be treated patients whose profiles are attenuated by therapy. The ordering of the clusters on apolipoprotein B, which took no part in the clustering, is the kind of internal consistency a descriptive partition should show, and no more.

### 4.3. Why Four, and What the Fifth Category Was

The first version of this analysis reported five clusters and was candid that most criteria preferred four; the fifth cluster, intermediate hyperglycaemia, was retained on a clinical argument about reassuring reports. The present analysis separates the two objectives that argument ran together. On the statistical objective, four clusters are the reproducible partition: every resampling criterion favours them, and only they survive whole-pipeline refitting and de novo re-derivation in another cohort. On the operational objective, the fifth category turned out to change the form of the report—a category passport instead of a glucose flag—and not whether an intermediate glucose is reported: under the corrected reference table, no patient with a glucose of 6.1 mmol/L or above receives a reassuring passport under either configuration. What remained of the argument was a defect in the reference table, which the correction removed. The general lesson we would pass on is procedural: an argument about reports must be evaluated on delivered reports, because a deterministic rule layer downstream of a clustering can absorb the very difference the choice of *k* appears to make.

The intermediate-hyperglycaemia group is nevertheless real as a region of the continuum, and its instability is what a group occupying the interior of a smooth density must show: boundaries on all sides and no gap to anchor them. It is surfaced in the primary system not as a cluster but as a flag, and the natural follow-up—whether patients with a glucose between the reference limit and 7.0 mmol/L differ in HbA1c or in incident diabetes from their lipid-pattern neighbours—requires data this study does not have.

### 4.4. The Continuum, Uncertainty and the Choice of a Hard Partition

Internal indices disagree: all three retained components are unimodal, HDBSCAN finds no dense cores and the silhouette is modest (0.26): cardiometabolic laboratory profiles form a continuum, and any partition imposes a soft grid on it. We chose a hard K-means partition for operational reasons—a screening report must give the same patient the same category on repeated runs, and Gaussian mixtures assigned roughly half of all patients with a maximum posterior below 0.7—and we acknowledge that this is an argument about communication, not about validity. The mixture’s low confidence is an honest estimate of assignment ambiguity, and the primary system now reports that ambiguity per patient: the relative margin to the second-nearest centroid, which is below 0.25 for one patient in five, and the border flag. A report that carries a category without its margin overstates what the data say.

### 4.5. Preprocessing Choices

Two lessons about preprocessing follow from the sensitivity analyses. First, the scaling decision is the largest single lever in the pipeline—larger than the choice between PCA and the full feature space, which changes almost nothing, and larger than the inclusion of derived ratios. Robust scaling gives glucose twice the weight it has under variance standardisation, and that weight is what produces a hyperglycaemia-defined cluster; we present it as a design choice whose consequences are quantified, not as a clinically correct weighting, and recommend that clustering studies of laboratory panels report their scaling with the same prominence as their algorithm. Second, algebraically derived ratios such as the atherogenic coefficient add no information and redistribute 6–8% of assignments; they belong in the reporting layer, not the feature space. The same logic obliges authors to verify that no primary feature is itself derived, using the coefficient of determination of the putative feature on its constituents (≥0.999 for any deterministic equation, 0.909 here) rather than the width of a mass-balance residual. On that criterion, LDL-C in this cohort is not calculated; which assay produced it must be documented from laboratory records rather than inferred from the data.

### 4.6. From Labels to Reports: Reference Intervals, Decision Limits and Intended Use

The interpretation layer is a hybrid: clustering, reference-interval flags, boundary correction and flag-based reassignment. Its complete specification—decision table with per-rule counts, reference intervals, templates, combination rules and pseudocode—is now part of the record (Table 6; Supplementary Tables S11a–d). Its behaviour on the identifier-disjoint cohort under the frozen model is the only external check available; the rules were developed and evaluated on the same data and are not free of that circularity. Auditability does not establish clinical validity and the layer’s output is a comment on laboratory values, not a clinical interpretation: it does not know the patient’s history, medication, blood pressure or symptoms.

The flags-only baseline quantifies the division of labour and, equally, its dependence on the reference table. The intervals in use are population reference intervals, most of them round numbers in mg/dL; they describe a reference population and are wide relative to guideline decision limits. This is why interval-by-interval flagging reproduces every C0 and most C1 assignments but misses 70% of the triglyceride–HDL-C pattern, and why a single guideline rule covers more of that pattern than the table’s flags. The gap between a reference interval and a decision limit is exactly the space in which multivariate description adds something—and also why the layer’s intended use must be stated precisely. It is a reference-interval description with a pattern comment; it is not a screening or treatment-decision tool, and the reports say so. The one decision limit it now uses, 7.0 mmol/L for the reassignment into the marked-hyperglycaemia category, is sourced and its effect quantified. Whether the glucose flag itself should be set at the 5.6 mmol/L prediabetes threshold rather than the population limit is a decision about the table’s purpose that belongs to the laboratory; the 401 patients between the two limits show what it would change.

Before any clinical use, the safeguards are those that any laboratory comment requires. The generated text is reviewed by a laboratory physician before release. Discordant profiles (arithmetic conflicts, border cases with a HIGH glucose) are escalated. The wording recommends discussion with a physician rather than urgency. The comment reaches the patient with the report, through the ordering physician, and never as a stand-alone message. Repeat testing is handled by a defined rule: each order is interpreted on its own values, with no comparison to earlier results until longitudinal comparison has itself been validated. A prospective silent deployment, in which reports are generated but not released and compared with the laboratory physician’s own comment, is the appropriate next step.

### 4.7. Population and Selection

The cohort is a large sample from a laboratory network in Kazakhstan and, to our knowledge, it is the first cluster analysis of a lipid–glucose panel from Central Asia. It is not representative of the country or of primary care: 84% of records are purchasers of a promotional panel, the network’s collection points are unevenly represented and one regional centre is absent from the network extract for reasons the exports do not explain. Cluster prevalences among promotional and non-promotional purchasers were similar (C0 31.8% vs. 34.9%, C2 28.9% vs. 26.5%), which suggests that the discount did not select a different laboratory population, but the sample remains self-selected. We therefore make no statement about regional public health, and we would expect the prevalences—not the geometry—to differ in a primary-care population.

### 4.8. Limitations

The design is cross-sectional and contains no clinical outcomes, diagnoses, medication, anthropometry, blood pressure, HbA1c or follow-up; the clusters are assessed for reproducibility and transportability, not for prognostic value, and the labels are descriptions of centroid profiles. Fasting status is instructed but not verified, collection dates and times are not exported and each record is a single measurement, so the clusters describe single-measurement laboratory profiles and not persistent metabolic states; glucose and triglycerides are the analytes most affected [33] and also the ones that define C2 and C3. Identifiers are order numbers, so repeat orders by the same person cannot be excluded (upper bound 5.6%). Sex is binary by construction of the source system. The validation records are part of the pooled cohort on which the primary descriptive model was fitted; the only leakage-free assessment is the centres-to-network analysis of Section 3.6, whose training cohort is less than half of the pool.

Analytical heterogeneity is documented and unexplained. The Friedewald residual and the frequency of physically impossible lipid combinations differ between exports and, more strongly, between collection points. The assay, calibrator and control material are documented and common to the network (Section 2.1). But, the laboratory keeps no chronology of reagent lots and the exports do not record the performing laboratory or module, so the lot, module and calibration history behind individual records cannot be reconstructed. Part of the prevalence shift between the development and validation cohorts, and part of the between-site variation in cluster prevalence, may therefore be analytical rather than biological, and the transportability estimates should be read as a comparison of two data sources rather than two populations. The arithmetic screen’s tolerance of 0.8 mmol/L is ad hoc; the excess it detects is present in every export at tighter tolerances, so the screen removes only the most extreme impossible profiles, and records with a smaller excess remain in the analysis.

The interpretation rules were written and evaluated on the same data and were not prespecified; the boundary multiplier and the transportability benchmarks are post hoc exploratory choices, not prespecified validation criteria. The reference table is a population reference table, and three of its irregularities remain undecided. The sample is self-selected purchasers of a commercial panel from one network in one country. The partition is soft: one patient in five has an assignment margin below 0.25, and the primary model does not reproduce identically within every sex and age stratum, most visibly in men.

### 4.9. A Validation Roadmap

Outcome validation is the essential next step, and it can be specified. Within the network, the existing consent for research use of laboratory data permits linking cluster membership—and the continuous positions on the glycaemic and lipid axes, which the model also provides—to subsequent HbA1c results, repeat glucose measurements and repeat lipid panels, giving a first test of whether the intermediate glucose flag and the C3 category identify patients who go on to meet HbA1c-defined dysglycaemia. Linkage to the national electronic health record would add diagnoses of diabetes and cardiovascular disease, dispensing of lipid-lowering and glucose-lowering drugs, and mortality; the analysis should adjust for age, sex, blood pressure, smoking, body-mass index, renal function and baseline disease, compare the categories against SCORE2 [3] and guideline-based lipid classifications [7,34], and report discrimination, calibration, reclassification and decision-curve net benefit for any prognostic claim. Prospectively, a silent deployment against laboratory-physician comments would test the interpretation layer itself. Until such work is carried out, the system is exploratory and not ready for clinical decision support.

## 5. Conclusions

A routine five-analyte lipid–glucose panel supports a reproducible four-category descriptive partition of laboratory profiles: lower glucose and lipids; higher LDL-C and total cholesterol; higher triglycerides with lower HDL-C; marked hyperglycaemia with adverse lipids. The partition is stable under bootstrap resampling with and without refitting the pipeline. It transports as a frozen classifier to an identifier-disjoint cohort, with prevalence-shift point estimates within post hoc exploratory benchmarks (one confidence interval slightly crossing them), and it is re-derived independently there. A five-cluster alternative is not, and is reported only as a reporting configuration whose sole operational effect is to express an intermediate glucose as a category rather than as a flag. The clusters are laboratory-derived descriptive phenotypes, and the study is exploratory: their clinical meaning, prognostic value and utility have not been demonstrated and are the subject of the validation roadmap above.

Three practices we would recommend to others clustering laboratory panels follow from the sensitivity analyses. Report the scaling decision as prominently as the algorithm, since it is the largest lever in the pipeline. Keep algebraically derived ratios out of the feature space and verify that no primary feature is itself calculated. When a rule layer sits downstream of the clustering, evaluate arguments about the number of clusters on the reports the layer delivers, with the layer specified completely enough to be re-implemented. The interpretation layer described here is a laboratory comment, not a clinical decision tool; with its decision table, reference intervals and one decision limit on the record, it can be audited, recalibrated and, in a silent deployment, tested. Its reference intervals are internally repaired population reference intervals, not prospectively validated clinical decision thresholds, and its categories—marked hyperglycaemia included—name single-measurement laboratory patterns, not diagnoses.

## Figures and Tables

**Table 1:** Analyte distributions in the analysed cohort (n=21,832 laboratory-order records, one adult five-analyte profile each). All analytes in mmol/L.

Variable	Mean ± SD	Median	IQR
Glucose	5.55 ± 1.57	5.23	4.85–5.73
Total cholesterol	5.36 ± 1.30	5.29	4.46–6.15
Triglycerides	1.55 ± 1.17	1.27	0.91–1.81
HDL-cholesterol	1.37 ± 0.39	1.31	1.08–1.60
LDL-cholesterol	3.40 ± 1.10	3.34	2.62–4.08

**Table 2:** Internal validity indices for K-means over k=2–8 on the three-component PCA space. Higher silhouette and Calinski–Harabasz and lower Davies–Bouldin indicate better separation. The indices disagree with one another; together with the resampling criteria of Section 3.3, they do not identify a number of clusters, and among the candidates for a report, they favour four over five.

*k*	Inertia	Silhouette	Calinski–Harabasz	Davies–Bouldin
2	58,538	0.499	8059	1.003
3	43,380	0.272	9251	1.193
4	35,196	0.260	9293	1.125
5	31,308	0.258	8513	1.133
6	27,956	0.235	8150	1.147
7	25,657	0.226	7726	1.132
8	23,658	0.227	7444	1.122

**Table 3:** Median analyte profiles, sizes and composition of the four clusters (development cohort, n=21,832). Analytes in mmol/L; cluster indices C0–C3 are assigned by the deterministic relabelling rule and match Figure 3, Figure 4 and Figure 5. The labels describe laboratory patterns and do not indicate diagnosis, prognosis or treatment priority. Interquartile ranges of the analytes are provided in Supplementary Table S4c, the five-cluster configuration F0–F4 in Tables S4a–b and the age and sex composition in Table S12.

Cluster	Glucose	TC	TG	HDL-C	LDL-C	*n*	%	Age	Women
C0—Lower glucose and lipids	5.13	4.14	1.04	1.24	2.41	7051	32.3	49.2	55.8%
C1—Higher LDL-C/TC	5.02	5.83	1.04	1.63	3.77	7529	34.5	53.8	75.0%
C2—Higher TG, lower HDL-C	5.56	5.86	2.06	1.12	3.89	6220	28.5	54.5	47.8%
C3—Marked hyperglycaemia	9.93	5.28	2.10	1.09	3.21	1032	4.7	60.4	50.2%

**Table 4:** Transportability of the frozen centres-only classifier: cluster counts and proportions in the training cohort (two regional centres, n=10,450) and the projected identifier-disjoint network cohort (n=11,382) after Hungarian matching of centroids (identity mapping). Δ pp is the absolute difference in percentage points with a Newcombe 95% confidence interval; displacement is the largest matched-centroid displacement over the three axes in units of the training within-cluster SD; *P* from two-proportion *z*-tests, given for completeness. Post hoc exploratory operational benchmarks: 5 percentage points and 0.5 SD (Supplementary Table S10b); the C1 interval crosses the five-point benchmark.

Cluster	Training *n* (%)	Validation *n* (%)	Δ pp (95% CI)	*p*	Displacement (SD)
C0—Lower glucose and lipids	3414 (32.7)	3619 (31.8)	−0.87 (−2.11 to +0.37)	0.17	0.305
C1—Higher LDL-C/TC	3574 (34.2)	3452 (30.3)	−3.87 (−5.11 to −2.63)	$9\times 10^{-10}$	0.345
C2—Higher TG, lower HDL-C	3020 (28.9)	3616 (31.8)	+2.87 (+1.65 to +4.09)	$4\times 10^{-6}$	0.204
C3—Marked hyperglycaemia	442 (4.2)	695 (6.1)	+1.88 (+1.29 to +2.46)	$5\times 10^{-10}$	0.141

**Table 5:** Sensitivity analyses: alternative feature sets, transformations, scaling, reduction and cohort definition, each compared with the primary four-cluster model (Hungarian label alignment; agreement = share of patients keeping their cluster). AC: atherogenic coefficient; AIP: atherogenic index of plasma. Glucose share: share of glucose in the total scaled variance of the feature space in which the model is fitted.

Model	Silhouette	Agreement	ARI vs. Primary	Glucose Share
Primary: 5 analytes, log, robust scaling, PCA(3)	0.260	—	—	40.7%
K-means in the full 5-D scaled space (no PCA)	0.230	98.5%	0.958	40.7%
+ AC =(TC−HDL)/HDL	0.261	91.6%	0.769	—
+ AIP $=\log_{10}\left(\mathrm{TG}/\mathrm{HDL}\right)$	0.266	93.7%	0.823	—
− LDL-C (four features)	0.255	89.2%	0.708	—
log, variance standardization (z-score)	0.290	91.8%	0.786	20.0%
log, no scaling	0.261	69.9%	0.452	11.9%
no log, robust scaling	0.274	68.2%	0.364	48.4%
no log, z-score	0.270	70.5%	0.373	20.0%
Tails trimmed at the 0.5/99.5 percentiles (n=20,985)	0.267	95.0%	0.880	—

**Table 6:** The reconciliation layer by stage and cohort. Counts and shares of records in the pooled development cohort under the primary model, and rates per 1000 records in the training and validation cohorts under the frozen centres-only model. A record moved by both stages is counted in each stage; the net row counts records whose delivered category differs from the K-means label. Per-rule counts are in Supplementary Table S11a.

Quantity	Development (Pooled), n=21,832	Training (Centres), n=10,450	Validation (Network), n=11,382
Stage 1, boundary correction (7 rules)	601 (2.8%)	27.9 per 1000	36.9 per 1000
Stage 2, flag-based correction (9 rules)	1210 (5.5%)	50.8 per 1000	47.5 per 1000
Moved by both stages	24	—	—
Delivered category ≠ K-means label	1787 (8.2%)	7.8%	8.3%
C0, K-means → delivered (%)	32.3 → 37.4	32.7 → 37.3	31.8 → 36.2
C1, K-means → delivered (%)	34.5 → 31.9	34.2 → 32.3	30.3 → 29.8
C2, K-means → delivered (%)	28.5 → 25.3	28.9 → 25.6	31.8 → 27.3
C3, K-means → delivered (%)	4.7 → 5.4	4.2 → 4.7	6.1 → 6.7

## Data Availability

The analysis code, unit tests, serialized model artifacts and the scripts that regenerate every table and figure of this article are deposited at Zenodo as a restricted-access record (DOI: 10.5281/zenodo.22064635; version 1.1.0 corresponds to this revision, and the SHA-256 checksum of the open-format parameter file centroids.json that Supplementary Table S22 reproduces is 5e4d5837ef6b8dd0ccc8f75d6d4ad2b5d89cf7b341c103596173f1081318fa48). The software is the proprietary work of CDL Olymp and is not openly licensed; access for verification is granted to editors and reviewers on request through the record, and to other researchers under a written agreement with the copyright holder. To allow independent re-implementation without access to the code, the complete reconciliation algorithm is given as a decision table and pseudocode in the Supplementary Material (Table S11a, Section S23), together with the reference intervals (Table S19), the serialised transform parameters and centroids (Table S22), the report templates and combination rules (Appendix B and Appendix C) and all aggregate outputs. The patient-level data are not publicly available due to patient privacy and institutional restrictions; de-identified data may be made available by the corresponding author on reasonable request and with the relevant approvals.

## Supplementary Materials

The following supporting information can be downloaded at https://www.mdpi.com/article/10.3390/diagnostics16183025/s1: Table S1: Cohort selection flow; Table S2: Full PCA loading matrix; Table S3: Per-feature influence; Table S4: The five-cluster reporting configuration (F0–F4); Table S5: Gap statistic and prediction strength; Table S6: Bootstrap cluster-wise stability; Table S7: Hartigan dip tests of unimodality; Table S8: LDL-C provenance—Friedewald residuals by export and by collection site; Table S9: Sensitivity models; Table S10: Transportability and de novo re-derivation; Table S11: The interpretation layer—decision table, reassignments and baselines; Table S12: Age and sex composition; sex- and age-stratified analysis; Table S13: Arithmetic-consistency failures by source export; Table S14: Per-feature transport statistics; Table S15: Flags fired at the cluster median profiles; Table S16: Per-patient assignment margin; Table S17: Analytes not used in clustering, by cluster; Table S18: Collection sites; Table S19: Reference intervals—corrected table (used for all primary results) and deployed table (before this study); Table S20: Data dictionary; Table S21: Worked patient-level examples; Table S22: Serialized parameters of the frozen model; Section S23: Pseudocode of the interpretation layer; STROBE checklist (cross-sectional studies).

## Abbreviations

The following abbreviations are used in this manuscript:

AC	Atherogenic coefficient, (TC−HDL-C)/HDL-C
AIP	Atherogenic index of plasma, $\log_{10}\left(\mathrm{TG}/\mathrm{HDL}\text{-}C\right)$
ARI	Adjusted Rand index
BIC	Bayesian information criterion
GMM	Gaussian mixture model
HDL-C	High-density-lipoprotein cholesterol
IQR	Interquartile range
KS	Kolmogorov–Smirnov
LDL-C	Low-density-lipoprotein cholesterol
PCA	Principal component analysis
SD	Standard deviation
TC	Total cholesterol
TG	Triglycerides

## Appendix A. Reference Intervals of the Interpretation Layer

Table A1 prints the age- and sex-specific reference intervals against which every analyte is flagged, in the corrected form used for all primary results; the two glucose rows that differ from the previously deployed table are marked, and the deployed values are given in Supplementary Table S19. The intervals are population reference intervals adopted in the network’s reporting system before this study. Their provenance is visible in their values: expressed in conventional units, 44 of the 50 limits of the deployed table (the rows of Table A1 with the deployed glucose values) fall within 0.5 mg/dL of a multiple of five (HDL-C 12 of 12, LDL-C 19 of 20, glucose 7 of 8, total cholesterol 6 of 8; the two triglyceride limits are the exception). That is the signature of a table authored in mg/dL and converted to SI units. Their source document, reference population, analytical method and fasting conditions are not recorded in the reporting system, and we were unable to identify the source document. They are *not* guideline decision limits or treatment targets: an “LDL-C NORMAL” flag does not imply that a guideline LDL-C goal is met, and the generated reports state this. Age is used in fractional years, and the upper bound of every age band is inclusive (a band “61–70” covers 60<age≤70). Pediatric bands are retained for inference completeness although the cohort is adults only. Flags for decreased values are informational; only HIGH flags and a LOW HDL-C flag participate in the reconciliation rules of Appendix D.

**Table A1:** Age- and sex-specific reference intervals used by the interpretation layer, corrected table (mmol/L; AC dimensionless). M: male; F: female; “any” applies to both sexes. † differs from the deployed table (deployed values: glucose 61–70, 4.44–6.38; glucose >70, 4.61–6.10). ‡ The fall-through row of the AC table covers every combination not listed—men under 20, 30–39 and 60 or older, women under 20 and over 30 and fractional ages between the listed integer bounds (e.g., 29.5)—so that coverage is complete; the three specific rows are the only sex- and age-specific AC limits the table carries (Section 3.9; Supplementary Table S19).

Analyte	Sex, Age Band (Years)	Lower	Upper
Glucose	any, <14	3.33	5.55
Glucose	any, 14–60	3.89	5.83
Glucose	any, 61–70	3.89 †	6.10 †
Glucose	any, >70	3.89 †	6.10
TC	any, <1	1.37	3.50
TC	any, 1–13	3.11	5.18
TC	any, 14–17	3.11	5.44
TC	any, ≥18	3.63	5.20
TG	any, ≤66	0.00	2.30
TG	any, >66	0.00	3.70
HDL-C	M, <20	0.78	1.68
HDL-C	M, ≥20	0.78	1.81
HDL-C	F, <20	0.78	1.68
HDL-C	F, 20–30	0.78	1.94
HDL-C	F, 31–40	0.78	2.07
HDL-C	F, >40	0.78	2.20
LDL-C	M, ≤29	1.55	4.53
LDL-C	M, 30–39	2.07	4.92
LDL-C	M, 40–49	2.33	5.31
LDL-C	M, 50–70	2.33	5.57
LDL-C	M, >70	2.33	4.92
LDL-C	F, ≤40	1.81	4.40
LDL-C	F, 41–50	2.07	4.92
LDL-C	F, 51–60	2.33	5.70
LDL-C	F, 61–70	2.59	6.09
LDL-C	F, >70	2.46	5.57
AC	M, 20≤age≤29	0.0	2.5
AC	M, 40≤age≤59	0.0	3.5
AC	F, 20≤age≤30	0.0	2.2
AC	all other sex–age combinations ‡	0.0	3.0

## Appendix B. Reference-Interval Flags and Single-Deviation Report Templates

Each analyte value is compared with its reference interval (Appendix A) and assigned a directional flag: HIGH, LOW, NORMAL, or MISSING. When a profile shows a single isolated deviation against an otherwise normal background, the report is generated from one of thirteen single-deviation templates (Table A2). Template T7 is reserved for arithmetically inconsistent results (Appendix D). Two dependency-pruning rules prevent redundant flags from reaching the report: the TC flag is suppressed when it is concordant with the LDL-C flag, and the AC flag when it is discordant with the HDL-C flag. When more than one independent deviation remains, templates are prioritised in the order glucose > TG > LDL-C > HDL-C > TC > AC. The priority applies to patients whose delivered category is C0; patients in C2 or C3 always receive the category passport with their flags listed, and patients in C1 receive the passport when two or more flags remain and a single-deviation template otherwise (Supplementary Section S23, step 12). Template wording describes the laboratory finding and recommends discussion with a physician; it does not state urgency, diagnosis or treatment.

**Table A2:** Thirteen single-deviation report templates of the interpretation layer (T1–T13).

Template	Analyte	Direction	Content
T1	Triglycerides	High	Raised triglycerides; lifestyle and metabolic context
T2	Glucose	High	Raised glucose; repeat fasting measurement or HbA1c suggested
T3	Total cholesterol	High	Raised total cholesterol
T4	HDL-cholesterol	Low	Reduced HDL-cholesterol
T5	LDL-cholesterol	High	Raised LDL-cholesterol
T6	AC	High	Raised atherogenic coefficient
T7	—	—	Arithmetic inconsistency (see Appendix D)
T8	Triglycerides	Low	Low triglycerides; informational
T9	Glucose	Low	Low glucose; clinical assessment suggested
T10	Total cholesterol	Low	Low total cholesterol; exclusion of systemic causes suggested
T11	HDL-cholesterol	High	Raised HDL-cholesterol; informational
T12	LDL-cholesterol	Low	Low LDL-cholesterol; informational
T13	AC	Low	Low atherogenic coefficient; informational

## Appendix C. Multi-Analyte Combination Rules

Eight combination rules encode co-occurring flag patterns and the laboratory pattern they resemble (Table A3). Rules are evaluated on the flagged analytes in the fixed order COM-1 to COM-8; “any lipid HIGH” denotes a HIGH flag on any of TC, LDL-C, TG or AC, and “any other HIGH” denotes a HIGH flag on any analyte other than the one named. The rules are non-exclusive and partially redundant (COM-7 subsumes COM-1, COM-2 and COM-4; COM-3 largely follows the LDL-C flag), because they select narrative fragments rather than categories: all satisfied rules are listed on the report, and none changes the category.

**Table A3:** Eight multi-analyte combination rules and the laboratory pattern each names.

Rule	Trigger Condition	Pattern Named
COM-1	TG High AND HDL-C Low	Raised TG with low HDL-C
COM-2	TG High AND glucose High	Raised TG with raised glucose
COM-3	LDL-C High AND AC High	Raised LDL-C
COM-4	TG High AND TC High	Raised TG with raised TC
COM-5	TC High AND TG normal	Isolated raised TC
COM-6	glucose High AND any lipid High	Raised glucose with a lipid deviation
COM-7	TG High AND any other analyte High	Raised TG with another deviation
COM-8	LDL-C High AND HDL-C Low	Raised LDL-C with low HDL-C

## Appendix D. Cluster–Flag Reconciliation Rules

The K-means label is reconciled with the flags in two stages. Stage 1, the *boundary correction*, applies only to border cases—patients whose distance to the assigned centroid exceeds 1.5 times that cluster’s median assignment distance—and reassigns them when their flags indicate a neighbouring pattern. Stage 2, the *flag-based correction*, applies to every patient and is iterated to a fixed point (at most three passes). Table A4 lists the sixteen reconciliation rules of the primary model—seven boundary-correction rules of stage 1 and nine flag-based rules of stage 2—followed, as a separate row, by the arithmetic-consistency check, which does not move a record between categories but replaces the report template. Rules of one source category are evaluated top-down and the first that fires wins. “Glucose HIGH^∗^” denotes the HIGH flag together with a glucose value of at least 7.0 mmol/L, the fasting-plasma-glucose threshold for diabetes [30] and the only decision limit the layer uses. A HIGH glucose flag below that value does not move a patient between categories and is reported as template T2 or alongside the category passport. The number of records each rule moved in the pooled development cohort, and the rate per 1000 records in the training and validation cohorts under the frozen centres-only model, are in Supplementary Table S11a, and the pseudocode of the whole layer in Supplementary Section S23. The arithmetic-consistency check that triggers template T7 fires when total cholesterol falls below the screen of Section 2.2 (TC<LDL-C+HDL-C−0.8) or when a recorded atherogenic coefficient differs from its recomputed value by more than 2.0.

**Table A4:** The complete reconciliation rule set of the primary model: seven stage-1 (boundary-correction) rules, nine stage-2 (flag-based) rules and, as a separate row, the arithmetic-consistency check, which replaces the report template without moving a record between categories. “Glucose HIGH^∗^”: the HIGH flag together with a glucose value of at least 7.0 mmol/L. Rule identifiers match the code and Supplementary Table S11a. The two italicised rows are stage headings that group the rules below them; they are not rules and carry no data.

Rule	From	Condition (Flags)	To
*Stage 1—border cases only*			
B4-01	C0	glucose HIGH^∗^ AND TG HIGH	C3
B4-02	C0	TC HIGH AND TG HIGH	C2
B4-03	C0	TC HIGH	C1
B4-04	C1	TG HIGH AND HDL-C LOW	C2
B4-05	C2	glucose HIGH^∗^ AND TG HIGH	C3
B4-06	C2	TG not HIGH AND HDL-C not LOW	C1
B4-07	C3	glucose not HIGH	C2
*Stage 2—every patient, iterated to a fixed point*			
R4-01	C0	glucose HIGH^∗^ AND (TG HIGH OR LDL-C HIGH)	C3
R4-02	C0	TC HIGH AND TG not HIGH	C1
R4-03	C0	TC HIGH AND TG HIGH	C2
R4-04	C0	TG HIGH AND HDL-C LOW	C2
R4-05	C1	glucose HIGH^∗^ AND (TG HIGH OR LDL-C HIGH)	C3
R4-06	C1	no flag at all	C0
R4-07	C2	glucose HIGH^∗^ AND (TG HIGH OR LDL-C HIGH)	C3
R4-08	C2	no flag at all	C0
R4-09	C3	glucose not HIGH	C2
Any	any	arithmetic inconsistency	Template T7

## References

[1] Roth G.A., Mensah G.A., Johnson C.O., Addolorato G., Ammirati E., Baddour L.M., Barengo N.C., Beaton A.Z., Benjamin E.J., Benziger C.P. (2020). Global Burden of Cardiovascular Diseases and Risk Factors, 1990–2019: Update From the GBD 2019 Study. J. Am. Coll. Cardiol..

[2] Visseren F.L.J., Mach F., Smulders Y.M., Carballo D., Koskinas K.C., Bäck M., Benetos A., Biffi A., Boavida J.-M., Capodanno D. (2021). 2021 ESC Guidelines on Cardiovascular Disease Prevention in Clinical Practice. Eur. Heart J..

[3] SCORE2 Working Group, ESC Cardiovascular Risk Collaboration (2021). SCORE2 Risk Prediction Algorithms: New Models to Estimate 10-Year Risk of Cardiovascular Disease in Europe. Eur. Heart J..

[4] Goff D.C., Lloyd-Jones D.M., Bennett G., Coady S., D’Agostino R.B., Gibbons R., Greenland P., Lackland D.T., Levy D., O’Donnell C.J. (2014). 2013 ACC/AHA Guideline on the Assessment of Cardiovascular Risk. Circulation.

[5] Ozarda Y. (2016). Reference Intervals: Current Status, Recent Developments and Future Considerations. Biochem. Medica.

[6] Alberti K.G.M.M., Eckel R.H., Grundy S.M., Zimmet P.Z., Cleeman J.I., Donato K.A., Fruchart J.C., James W.P., Loria C.M., Smith S.C. (2009). Harmonizing the Metabolic Syndrome: A joint interim statement of the International Diabetes Federation Task Force on Epidemiology and Prevention; National Heart, Lung, and Blood Institute; American Heart Association; World Heart Federation; International Atherosclerosis Society; and International Association for the Study of Obesity. Circulation.

[7] Mach F., Baigent C., Catapano A.L., Koskinas K.C., Casula M., Badimon L., Chapman M.J., De Backer G.G., Delgado V., Ference B.A. (2020). 2019 ESC/EAS Guidelines for the Management of Dyslipidaemias: Lipid modification to reduce cardiovascular risk. Eur. Heart J..

[8] Shah S.J., Katz D.H., Selvaraj S., Burke M.A., Yancy C.W., Gheorghiade M., Bonow R.O., Huang C.-C., Deo R.C. (2015). Phenomapping for Novel Classification of Heart Failure with Preserved Ejection Fraction. Circulation.

[9] Ahmad T., Pencina M.J., Schulte P.J., O’Brien E., Whellan D.J., Piña I.L., Kitzman D.W., Lee K.L., O’Connor C.M., Felker G.M. (2014). Clinical Implications of Chronic Heart Failure Phenotypes Defined by Cluster Analysis. J. Am. Coll. Cardiol..

[10] Antman E.M., Loscalzo J. (2016). Precision Medicine in Cardiology. Nat. Rev. Cardiol..

[11] Hubert L., Arabie P. (1985). Comparing Partitions. J. Classif..

[12] Dobiášová M., Frohlich J. (2001). The Plasma Parameter log(TG/HDL-C) as an Atherogenic Index. Clin. Biochem..

[13] Friedewald W.T., Levy R.I., Fredrickson D.S. (1972). Estimation of the Concentration of Low-Density-Lipoprotein Cholesterol in Plasma, without Use of the Preparative Ultracentrifuge. Clin. Chem..

[14] Martin S.S., Blaha M.J., Elshazly M.B., Toth P.P., Kwiterovich P.O., Blumenthal R.S., Jones S.R. (2013). Comparison of a Novel Method vs the Friedewald Equation for Estimating Low-Density Lipoprotein Cholesterol Levels from the Standard Lipid Profile. JAMA.

[15] Sampson M., Ling C., Sun Q., Harb R., Ashmaig M., Warnick R., Sethi A., Fleming J.K., Otvos J.D., Meeusen J.W. (2020). A New Equation for Calculation of Low-Density Lipoprotein Cholesterol in Patients with Normolipidemia and/or Hypertriglyceridemia. JAMA Cardiol..

[16] Pedregosa F., Varoquaux G., Gramfort A., Michel V., Thirion B., Grisel O., Blondel M., Prettenhofer P., Weiss R., Dubourg V. (2011). Scikit-learn: Machine Learning in Python. J. Mach. Learn. Res..

[17] Millán J., Pintó X., Muñoz A., Zúñiga M., Rubiés-Prat J., Pallardo L.F., Masana L., Mangas A., Hernández-Mijares A., González-Santos P. (2009). Lipoprotein Ratios: Physiological Significance and Clinical Usefulness in Cardiovascular Prevention. Vasc. Health Risk Manag..

[18] Jolliffe I.T., Cadima J. (2016). Principal Component Analysis: A Review and Recent Developments. Philos. Trans. R. Soc. A.

[19] MacQueen J. (1967). Some Methods for Classification and Analysis of Multivariate Observations. Proceedings of the Fifth Berkeley Symposium on Mathematical Statistics and Probability.

[20] McLachlan G.J., Peel D. (2000). Finite Mixture Models.

[21] Campello R.J.G.B., Moulavi D., Sander J. (2013). Density-Based Clustering Based on Hierarchical Density Estimates. Advances in Knowledge Discovery and Data Mining (PAKDD 2013).

[22] Rousseeuw P.J. (1987). Silhouettes: A Graphical Aid to the Interpretation and Validation of Cluster Analysis. J. Comput. Appl. Math..

[23] Caliński T., Harabasz J. (1974). A Dendrite Method for Cluster Analysis. Commun. Stat..

[24] Davies D.L., Bouldin D.W. (1979). A Cluster Separation Measure. IEEE Trans. Pattern Anal. Mach. Intell..

[25] Tibshirani R., Walther G., Hastie T. (2001). Estimating the Number of Clusters in a Data Set via the Gap Statistic. J. R. Stat. Soc. Ser. B.

[26] Tibshirani R., Walther G. (2005). Cluster Validation by Prediction Strength. J. Comput. Graph. Stat..

[27] Hennig C. (2007). Cluster-Wise Assessment of Cluster Stability. Comput. Stat. Data Anal..

[28] Hartigan J.A., Hartigan P.M. (1985). The Dip Test of Unimodality. Ann. Stat..

[29] American Diabetes Association (2021). Classification and Diagnosis of Diabetes: Standards of Medical Care in Diabetes—2021. Diabetes Care.

[30] American Diabetes Association Professional Practice Committee (2026). Diagnosis and Classification of Diabetes: Standards of Care in Diabetes—2026. Diabetes Care.

[31] von Elm E., Altman D.G., Egger M., Pocock S.J., Gøtzsche P.C., Vandenbroucke J.P., for the STROBE Initiative (2007). The Strengthening the Reporting of Observational Studies in Epidemiology (STROBE) Statement: Guidelines for Reporting Observational Studies. Lancet.

[32] Collins G.S., Moons K.G.M., Dhiman P., Riley R.D., Beam A.L., Van Calster B., Ghassemi M., Liu X., Reitsma J.B., van Smeden M. (2024). TRIPOD+AI Statement: Updated Guidance for Reporting Clinical Prediction Models That Use Regression or Machine Learning Methods. BMJ.

[33] Nordestgaard B.G., Langsted A., Mora S., Kolovou G., Baum H., Bruckert E., Watts G.F., Sypniewska G., Wiklund O., Borén J. (2016). Fasting Is Not Routinely Required for Determination of a Lipid Profile: Joint Consensus Statement from the EAS and EFLM. Eur. Heart J..

[34] Mach F., Koskinas K.C., Roeters van Lennep J.E., Tokgözoğlu L., Badimon L., Baigent C., Benn M., Binder C.J., Catapano A.L., De Backer G.G. (2025). 2025 Focused Update of the 2019 ESC/EAS Guidelines for the Management of Dyslipidaemias. Eur. Heart J..

