# Supplementary material for: Unsupervised Descriptive Phenotyping of Cardiometabolic Laboratory Patterns from a Routine Five-Analyte Panel: Stability, Transportability and a Rule-Based Laboratory Interpretation Layer
Source: Diagnostics (Basel). 2026 Sep 18;16(18):3025. doi: 10.3390/diagnostics16183025 (PMC13605647; doi:10.3390/diagnostics16183025)
Supplement: Supplementary file 1 [file diagnostics-16-03025-s001.zip › diagnostics-4550369-supplementary.pdf]

Supplementary Materials

Unsupervised Descriptive Phenotyping of Cardiometabolic Laboratory Patterns from a Routine Five-Analyte Panel: Stability, Transportability and a Rule-Based Laboratory Interpretation Layer

Yerlan Suleimenov, Ayat Assemov, Bakhytzhan Seksenbayev, Akezhan Koishybay, Albina Omarova, Baurzhan Nurlan

All quantities in this document are generated by the analysis scripts (`reproduce_paper.py`, `analysis_*.py`) from the raw exports and read from their machine-readable outputs (`outputs/summary.json`, `analysis_*.json`, `tables/*.csv`); no value is typed by hand. Cluster labels: C0–C3 denote the primary four-cluster model (C0 lower glucose and lipids; C1 higher LDL-C/TC; C2 higher TG, lower HDL-C; C3 marked hyperglycaemia with adverse lipids); F0–F4 denote the five-cluster reporting configuration, in which F0–F3 correspond to C0–C3 by the same naming rule and F4 is the additional intermediate-hyperglycaemia cluster.

Contents

S1 Cohort selection flow 2

S2 PCA loadings 2

S3 Per-feature influence 3

S4 Five-cluster configuration 3

S5 Gap statistic and prediction strength 3

S6 Bootstrap stability 4

S7 Dip tests 5

S8 LDL-C provenance 5

S9 Sensitivity models 6

S10 Transportability 7

S11 Interpretation layer 8

S12 Age and sex 10

S13 Arithmetic failures 10

S14 Per-feature transport 10

S15 Flags at medians 11

S16 Assignment margin 11

S17 Analytes not used in clustering 11

S18 Collection sites 12

S19 Reference intervals 13

S20 Data dictionary 14

S21 Worked examples 15

S22 Model parameters 16

S23 Pseudocode 16

STROBE checklist 17

Table S1. Cohort selection flow

Table S1: Sequential cohort selection. The development (regional centres) and identifier-disjoint validation sub-cohorts receive the same filters independently.

Step	Remaining n	Removed
Pooled: records loaded	28,124	0
Pooled: unique ids	22,328	5,796
Pooled: adults 18 100	21,885	443
Pooled: plausible values	21,872	13
Pooled: arithmetic consistent	21,850	22
Pooled: complete cases	21,832	18
Centres: records loaded	10,716	0
Centres: unique ids	10,716	0
Centres: adults 18 100	10,465	251
Centres: plausible values	10,456	9
Centres: arithmetic consistent	10,456	0
Centres: complete cases	10,450	6
Network-unique: records loaded	11,612	0
Network-unique: unique ids	11,612	0
Network-unique: adults 18 100	11,420	192
Network-unique: plausible values	11,416	4
Network-unique: arithmetic consistent	11,394	22
Network-unique: complete cases	11,382	12
Missing values per analyte immediately before the complete-case step (STROBE 14b)		
Glucose	—	18
TC	—	1
TG	—	1
HDL-C	—	1
LDL-C	—	1
Rows with any missing analyte	21,850	18

The per-analyte counts sum to more than the number of incomplete rows because the gaps are nested: each missing lipid value occurs in a record that also lacks glucose, so the rows removed by the complete-case step are exactly the rows with a missing glucose. No value was imputed. Sex was recorded for every one of the 22,328 unique records (0 unknown after harmonisation of the numeric and textual encodings); the source system offers two values, and no intersex or non-binary category exists in it. 443 records had an age that was missing, unparseable or outside 18–100 years.

Table S2. Full PCA loading matrix

Table S2: Loadings of the three retained principal components (explained variance 45.4%, 29.2% and 18.2%; total 92.8%).

Analyte	PC1	PC2	PC3
Glucose	0.904	-0.165	-0.391
TC	0.096	0.682	-0.070
TG	0.359	0.207	0.657
HDL-C	-0.201	0.174	-0.638
LDL-C	0.067	0.659	-0.063

Clustering in the full five-dimensional scaled space instead of the three-component projection gives a partition with ARI 0.958 against the primary model and 98.5% identical assignments; silhouette of the primary partition in the PCA space 0.260, of the five-dimensional partition in its own space 0.230 (Table S9).

Table S3. Per-feature influence

Table S3: Share of each analyte in the total scaled variance and in the between-cluster separation (scaled feature space), with and without the marked-hyperglycaemia cluster C3.

Analyte	Scaled-variance share	Between-cluster share	Between-cluster share, C3 excluded
Glucose	40.7%	47.7%	7.3%
TC	14.4%	15.2%	28.2%
TG	17.3%	14.7%	23.9%
HDL-C	13.2%	8.9%	15.6%
LDL-C	14.4%	13.5%	25.0%

Table S4. The five-cluster reporting configuration (F0–F4)

Table S4: (a) Median analyte profiles (mmol/L), sizes and composition of the five-cluster configuration fitted on the same feature space as the primary model. F0–F3 are named by the same rule as C0–C3; F4 is the second-highest glucose centroid. ARI between the two partitions 0.767; the raw labels differ for 14.8% of the cohort.

Cluster	Pattern	Glucose	TC	TG	HDL-C	LDL-C	n	%
F0	Lower glucose & lipids	5.06	4.14	1.01	1.26	2.41	6,515	29.8
F1	Higher LDL-C/TC	5.04	5.83	1.02	1.66	3.76	6,910	31.7
F2	Higher TG, lower HDL-C	5.33	6.02	2.08	1.12	4.03	5,344	24.5
F3	Marked hyperglycaemia	11.89	5.54	2.39	1.06	3.32	609	2.8
F4	Intermediate hyperglycaemia	6.72	4.99	1.61	1.17	3.18	2,454	11.2

Table S4: (b) Cross-tabulation of the primary four-cluster labels (rows) against the five-cluster configuration (columns). The additional cluster F4 (2,454 patients, 11.2% of the cohort) is drawn from C0 24.9%, C1 3.9%, C2 54.0%, C3 17.2%.

	F0	F1	F2	F3	F4
C0	6,351	31	59	0	610
C1	152	6,819	463	0	95
C2	12	60	4,822	0	1,326
C3	0	0	0	609	423

Table S5. Gap statistic and prediction strength

Table S5: Gap statistic (20 uniform reference draws) and out-of-sample prediction strength over 15 random split-halves (mean ± SE across splits; the minimum over clusters is the criterion of Tibshirani & Walther, the mean over clusters is given alongside). The gap grid includes $k = 1$, the no-clustering null this statistic exists to test: the one-standard-error rule then selects $k = 1$, whereas the same rule restricted to $k \geq 2$ selects $k = 4$. On paired splits the $k = 5$ minus $k = 4$ difference in the minimum is -0.122 ± 0.052 ($t = -2.34$ on 14 degrees of freedom).

k	Gap	s_k	PS, min over clusters	PS, mean over clusters
1	2.301	0.004	—	—
2	2.027	0.005	0.912 ± 0.028	0.956 ± 0.014
3	2.158	0.005	0.913 ± 0.021	0.962 ± 0.008
4	2.197	0.004	0.898 ± 0.014	0.948 ± 0.006
5	2.184	0.004	0.776 ± 0.056	0.867 ± 0.038
6	2.158	0.005	0.694 ± 0.026	0.829 ± 0.016
7	2.127	0.004	—	—

Table S6. Bootstrap cluster-wise stability

Table S6: (a) Best-match Jaccard recovery of each cluster over 100 bootstrap resamples (Hennig 2007). Resamples of size n are drawn with replacement at the record level, duplicated records are retained as duplicates in fitting and evaluation, K-means (`n_init`=10) is refitted on each resample and every reference cluster is matched to the resample cluster with the largest Jaccard index (best match, no one-to-one constraint). "Fixed projection keeps the scaler and PCA of the full cohort; "pipeline refitted refits log–robust-scaler–PCA on every resample. Percentile 95% intervals; "dissolved is the share of resamples with Jaccard < 0.5.

Cluster	Scheme	Mean	Median	10th pct	95% interval	Dissolved
Primary model, $k = 4$						
C0	fixed projection	0.981	0.983	0.967	0.956–0.994	0%
C0	pipeline refitted	0.980	0.982	0.968	0.957–0.990	0%
C1	fixed projection	0.973	0.974	0.957	0.951–0.992	0%
C1	pipeline refitted	0.969	0.970	0.956	0.945–0.986	0%
C2	fixed projection	0.971	0.972	0.955	0.947–0.987	0%
C2	pipeline refitted	0.965	0.966	0.953	0.940–0.980	0%
C3	fixed projection	0.965	0.968	0.941	0.913–0.995	0%
C3	pipeline refitted	0.964	0.967	0.941	0.910–0.994	0%
Five-cluster configuration, $k = 5$						
F0	fixed projection	0.923	0.974	0.693	0.464–0.991	6%
F0	pipeline refitted	0.864	0.968	0.490	0.453–0.991	12%
F1	fixed projection	0.915	0.973	0.815	0.418–0.991	10%
F1	pipeline refitted	0.842	0.956	0.441	0.393–0.988	23%
F2	fixed projection	0.918	0.958	0.773	0.586–0.986	0%
F2	pipeline refitted	0.861	0.940	0.567	0.512–0.983	2%
F3	fixed projection	0.934	0.976	0.699	0.600–0.998	0%
F3	pipeline refitted	0.886	0.959	0.603	0.567–0.999	0%
F4	fixed projection	0.844	0.926	0.366	0.172–0.970	11%
F4	pipeline refitted	0.745	0.899	0.181	0.172–0.978	23%

Table S6: (b) Bayesian information criterion of the Gaussian mixtures by number of components and covariance structure (lower is better). The full-covariance minimum at $k = 5$ is the only criterion applied in this work that selects five components; the diagonal and tied variants select $k = 8$ and $k = 7$.

Covariance	$k = 2$	$k = 3$	$k = 4$	$k = 5$	$k = 6$	$k = 7$	$k = 8$
full	180,549	180,039	179,899	179,677	179,765	179,710	179,752
diag	183,093	181,991	180,680	180,114	180,178	180,066	179,770
tied	184,335	184,515	184,224	181,903	181,772	181,571	181,579

Table S6: (c) Multi-seed numerical reproducibility of the primary partition and of the delivered (post-reconciliation) category: adjusted Rand index against the reference seed.

Quantity	Mean ARI	Minimum ARI
K-means, 8 non-reference seeds	0.988	0.977
Gaussian mixture (tied), same seeds	0.987	0.982
Delivered category after reconciliation, 4 seeds	0.987	0.983

Table S7. Hartigan dip tests of unimodality

Table S7: Dip statistics and p-values for the retained principal components and the scaled analytes. The clustering space (PC1–PC3) is unimodal ($p > 0.99$ for each component). Three of the five analyte marginals do reject unimodality at $\alpha = 0.05$ — HDL-C, triglycerides and glucose, but not total cholesterol or LDL-C — as a mixed-sex screening population with a prediabetic shoulder would produce. The continuum claim of the article concerns the retained components, in which the clustering is performed, not the marginals.

Variable	Dip	p
PC1	0.00117	>0.99
PC2	0.00103	>0.99
PC3	0.00117	>0.99
Glucose	0.00426	0.0104
TC	0.00279	0.341
TG	0.00485	0.00168
HDL-C	0.00599	2.59e-05
LDL-C	0.00246	0.569

Table S8. LDL-C provenance: Friedewald residuals by export and by collection site

Table S8: (a) Residual r = TC − LDL − HDL − TG/2.2 per record. A Friedewald-calculated LDL-C would give $r \equiv 0$; instead the residual is wide, widens with TG, and LDL-C is reported even where calculation is conventionally suppressed (TG > 4.5 mmol/L). The R^2 of LDL-C on its fellow analytes excludes every deterministic equation ($R^2 \geq 0.999$ for Friedewald, Martin–Hopkins and Sampson); it does not identify the assay.

Quantity	Value
Residual mean (mmol/L)	-0.110
Residual SD (mmol/L)	0.366
Share $\|r\| < 0.01$	2.8%
Share $\|r\| < 0.10$	28.2%
Share $\|r\| < 0.20$	52.6%
Share within 0.015 mmol/L of the Friedewald value	4.3%
Residual SD, TG < 2.3 (n=18,804)	0.288
Residual SD, TG 2.3–4.5 (n=2,585)	0.505
Residual SD, TG > 4.5 (n=443)	1.119
R^2, linear LDL ∼ TC+HDL+TG	0.909
LDL-C missing among raw records with TG > 4.5	0.0% of 449
Stratified by source export	
Almaty (n=5,646): residual SD / R^2	0.314 / 0.933
Astana (n=4,804): residual SD / R^2	0.287 / 0.938
network-unique (n=11,382): residual SD / R^2	0.414 / 0.889
pooled (n=21,832): residual SD / R^2	0.366 / 0.909

Table S8: (b) The same residual by collection site (the two leading digits of the laboratory order number encode the collection point), for the 26 sites with at least 200 records. "Excess >0" is the share of records in which LDL-C + HDL-C exceeds total cholesterol by any amount; "failures are the records the deployed screen (tolerance 0.8 mmol/L) removed, counted before that filter. The spread across sites is the analytical heterogeneity the article reports as a limitation.

Site	Export	n	Resid. SD	R^2	Excess >0	Failures	Glucose median
70	almaty	4,806	0.298	0.939	3.2%	0	5.02
10	astana	4,130	0.282	0.939	2.5%	0	5.24
62	network	1,351	0.306	0.939	6.5%	0	5.15
61	network	1,157	0.261	0.964	4.7%	0	5.39
74	almaty	833	0.360	0.935	0.1%	0	5.04
37	network	721	0.183	0.979	5.5%	0	5.23
50	network	585	0.363	0.916	5.8%	1	5.31
24	network	539	0.457	0.943	2.0%	1	5.21
42	network	514	0.301	0.948	0.8%	0	5.07
94	network	450	0.251	0.959	0.2%	0	5.22
19	astana	432	0.326	0.950	4.6%	0	5.19
47	network	401	0.267	0.956	0.8%	0	5.55
90	network	348	0.535	0.770	1.1%	0	5.16
41	network	337	0.355	0.924	9.5%	6	5.65
52	network	329	0.413	0.873	10.3%	0	5.42
40	network	286	1.045	0.519	5.6%	7	5.56
35	network	244	0.394	0.897	3.3%	0	5.37
63	network	237	0.255	0.965	4.2%	0	5.49
93	network	227	0.207	0.970	1.3%	0	5.52
26	network	222	0.349	0.972	0.0%	0	5.49
48	network	217	0.237	0.973	0.5%	0	5.35
98	network	214	0.354	0.899	1.9%	0	5.46
92	network	212	0.218	0.954	8.0%	0	5.35
28	network	210	0.353	0.969	0.9%	0	5.39
75	network	205	0.318	0.925	6.3%	0	5.45
36	network	204	0.428	0.892	15.7%	0	5.41

Table S9. Sensitivity models

Table S9: Alternative feature sets, scaling and cohort definition versus the primary four-cluster model (Hungarian alignment; agreement = share of patients keeping their cluster). Glucose share = share of glucose in the total scaled variance of the feature space in which the alternative model is fitted.

Model	n	Silhouette	Agreement	ARI vs primary	Glucose share
Primary: 5 analytes, log, robust scaling, PCA(3)	21,832	0.26	—	—	40.7%
+ Atherogenic coefficient (6 features)	21,832	0.261	91.6%	0.769	—
+ AIP = $\log_{10}$(TG/HDL-C)	21,832	0.266	93.7%	0.823	—
− LDL-C (4 features; PCA 94.9%)	21,832	0.255	89.2%	0.708	—
log, variance standardization (z-score)	21,832	0.29	91.8%	0.786	20.0%
log, no scaling	21,832	0.261	69.9%	0.452	11.9%
no log, robust scaling	21,832	0.274	68.2%	0.364	48.4%
no log, z-score	21,832	0.27	70.5%	0.373	20.0%
K-means in the full 5-D scaled space (no PCA)	21,832	0.23	98.5%	0.958	—
Tails trimmed at 0.5/99.5 percentiles	20,985	0.267	95.0%	0.880	—

Cluster shares under trimming: 32.3% / 32.4% / 29.4% / 6.0% against 32.3% / 34.5% / 28.5% / 4.7% in the primary model; centroid glucose of C3 9.12 mmol/L after trimming. The marked-hyperglycaemia cluster is defined by extreme glucose, so removing the upper 0.5% of each analyte necessarily rebuilds it.

Table S10. Transportability and de novo re-derivation

Table S10: (a) Cluster prevalence, training (n=10,450) versus identifier-disjoint validation (n=11,382) cohort under the frozen centres-only model. Proportions carry Wilson 95% intervals and their difference a Newcombe 95% interval, in percentage points; the absolute difference is the primary quantity, z-test P values are given for completeness.

Cluster	Train % (95% CI)	Val. % (95% CI)	Δ pp (95% CI)	Rel. Δ	P
C0	32.7 (31.8–33.6)	31.8 (30.9–32.7)	-0.87 (-2.11 to +0.37)	-2.7%	0.17
C1	34.2 (33.3–35.1)	30.3 (29.5–31.2)	-3.87 (-5.11 to -2.63)	-11.3%	9.5e-10
C2	28.9 (28.0–29.8)	31.8 (30.9–32.6)	+2.87 (+1.65 to +4.09)	+9.9%	4.1e-06
C3	4.2 (3.9–4.6)	6.1 (5.7–6.6)	+1.88 (+1.29 to +2.46)	+44.4%	4.6e-10

Table S10: (b) Acceptability thresholds for transportability, declared before the verdicts were read but after the first submission (post hoc): a prevalence shift of at most 5 percentage points per cluster and a matched-centroid displacement of at most 0.5 within-cluster standard deviations on every principal axis.

Cluster	Δ pp	Passes	CI within threshold	Max displacement (SD)	Passes
C0	-0.87	yes	yes	0.305	yes
C1	-3.87	yes	no	0.345	yes
C2	+2.87	yes	yes	0.204	yes
C3	+1.88	yes	yes	0.141	yes

Table S10: (c) De novo re-derivation in the validation cohort, for the primary model and for the five-cluster configuration, and the no-population-shift reference. "Matched agreement aligns de novo clusters to development centroids by the Hungarian algorithm on standardized profiles before comparing labels.

Quantity	$k = 4$	$k = 5$
PCA axis congruence dev vs ext (|cos|, PC1/PC2/PC3)	0.995/0.993/0.999	same
Explained variance, ext PCA (3 comp.)	92.7%	same
ARI, frozen (projected) vs de novo labels on ext	0.815	0.328
Matched agreement, frozen vs de novo	93.1%	48.1%
Naming rule reproduced by centroid matching	yes	no
Glucose gap, 2nd vs 3rd centroid, dev / ext (mmol/L)	0.48 / 0.42	1.49 / 0.19
Reference: ARI of two half-cohort de novo models (dev)	0.950	—
Calinski–Harabasz on ext, k=3 / 4 / 5	5015 / 5005 / 4577	same

De novo prevalences on the matched correspondence, relative to the training cohort: $k = 4$ C0 -1.6%, C1 +1.3%, C2 -2.7%, C3 +20.1%; $k = 5$ F0 -35.8%, F1 -29.3%, F2 -4.9%, F3 +92.8%, F4 +161.2%.

Table S10: (d) Per-feature Wasserstein (log scale) and KS statistics between matched clusters, training vs validation.

Cluster	Feature	Wasserstein (log)	KS
C0	Glucose	0.0332	0.1483
C0	TC	0.0195	0.0661
C0	TG	0.0148	0.0372
C0	HDL-C	0.0138	0.0400
C0	LDL-C	0.0087	0.0367
C1	Glucose	0.0278	0.1417
C1	TC	0.0134	0.0530
C1	TG	0.0069	0.0234
C1	HDL-C	0.0127	0.0602
C1	LDL-C	0.0262	0.0752
C2	Glucose	0.0229	0.1079
C2	TC	0.0068	0.0332
C2	TG	0.0272	0.0460
C2	HDL-C	0.0140	0.0590
C2	LDL-C	0.0225	0.0544
C3	Glucose	0.0135	0.0410
C3	TC	0.0344	0.0880
C3	TG	0.0340	0.0429
C3	HDL-C	0.0086	0.0487
C3	LDL-C	0.0300	0.0677

Table S11. The interpretation layer: decision table, reassignments and baselines

Table S11: (a) Complete decision table of the two-stage reconciliation for the primary model, with the number of patients moved by each rule in the development cohort (pooled, n=21,832), and per 1,000 patients in the centres-only development cohort and the identifier-disjoint validation cohort under the frozen centres-only model. Rules of one source cluster are evaluated top-down and the first that fires wins; stage 1 applies only to border cases (assignment distance $>$ 1.5 $\times$ the cluster median), stage 2 to every patient and is iterated to a fixed point (at most three passes). "glucose HIGH* means the HIGH flag together with a glucose value of at least 7.0 mmol/L, the single guideline decision limit used by the layer.

Rule	Stage	From	Condition → To	Pooled n	Dev /1000	Val /1000
B4-01	border	C0	glucose HIGH* and TG HIGH → C3	5	0.0	0.3
B4-02	border	C0	TC HIGH and TG HIGH → C2	1	0.0	0.1
B4-03	border	C0	TC HIGH → C1	3	0.3	0.0
B4-04	border	C1	TG HIGH and HDL-C LOW → C2	0	0.0	0.0
B4-05	border	C2	glucose HIGH* and TG HIGH → C3	109	4.0	3.9
B4-06	border	C2	TG not HIGH and HDL-C not LOW → C1	483	23.6	32.7
B4-07	border	C3	glucose not HIGH → C2	0	0.0	0.0
R4-01	flag	C0	glucose HIGH* and (TG HIGH or LDL-C HIGH) → C3	0	0.0	0.0
R4-02	flag	C0	TC HIGH and TG not HIGH → C1	5	0.4	0.2
R4-03	flag	C0	TC HIGH and TG HIGH → C2	0	0.0	0.0
R4-04	flag	C0	TG HIGH and HDL-C LOW → C2	26	1.1	0.7
R4-05	flag	C1	glucose HIGH* and (TG HIGH or LDL-C HIGH) → C3	24	0.9	1.2
R4-06	flag	C1	no flag at all → C0	1023	42.6	36.9
R4-07	flag	C2	glucose HIGH* and (TG HIGH or LDL-C HIGH) → C3	9	0.1	0.1
R4-08	flag	C2	no flag at all → C0	123	5.8	8.4
R4-09	flag	C3	glucose not HIGH → C2	0	0.0	0.0

Table S11: (b) Cluster distribution before (K-means label) and after (delivered category) reconciliation, and the transition matrix (rows: K-means label; columns: delivered category).

Cluster	K-means n (%)	Delivered n (%)		
C0	7,051 (32.3)	8,157 (37.4)		
C1	7,529 (34.5)	6,973 (31.9)		
C2	6,220 (28.5)	5,523 (25.3)		
C3	1,032 (4.7)	1,179 (5.4)		
Transition	C0	C1	C2	C3
C0	7,011	8	27	5
C1	1,023	6,506	0	0
C2	123	459	5,496	142
C3	0	0	0	1,032

Stage 1 moved 601 patients (2.75%), stage 2 1,210 (5.54%); 24 were moved by both stages, so the net number of patients whose delivered category differs from their K-means label is 1,787 (8.19%). 18,024 patients (82.56%) carry at least one reference-interval flag.

Table S11: (c) Flags-only baseline (reconciliation rules with no cluster input, every patient started in C0) versus the full system, and the sensitivity of the boundary-correction threshold.

Quantity	Value
Overall agreement with the full system	76.9%
ARI	0.542
Recall of delivered C0 by flags alone	100.0%
Recall of delivered C1 by flags alone	92.0%
Recall of delivered C2 by flags alone	30.3%
Recall of delivered C3 by flags alone	45.5%
Boundary threshold (multiples of the median assignment distance)	
1.25: labels changed vs K-means / agreement with 1.5	10.16% / 98.03%
1.5: labels changed vs K-means / agreement with 1.5	8.19% / 100.0%
1.75: labels changed vs K-means / agreement with 1.5	7.1% / 98.91%
2.0: labels changed vs K-means / agreement with 1.5	6.46% / 98.27%

Table S11: (d) Effect of the glucose decision limit on transfers into the marked-hyperglycaemia cluster C3. “Flag-only is the rule set in routine use before this revision (any HIGH glucose flag qualified).

Quantity	With 7.0 mmol/L limit (adopted)	Flag-only
K-means C3, n	1,032	1,032
Delivered C3, n	1,179	1,729
Moved into C3 by rules, n	147	697
Median glucose of those moved in (mmol/L)	7.34	6.39
Share of those moved in with glucose $\geq$ 7.0	100.0%	21.1%
Moved in with glucose $<$ 6.1 mmol/L, n	0	184
Net delivered categories changed vs K-means	8.19%	10.53%
Patients whose delivered category differs between the two variants		550
Where they stay under the adopted rules		C2 491, C1 44, C0 15

Table S12. Age and sex composition; sex- and age-stratified analysis

Table S12: (a) Median age (IQR) and share of women per cluster (development cohort; women 59.9% of the cohort), with Wilson 95% intervals.

Cluster	Pattern	n	Age, median (IQR)	Women (95% CI)
C0	Lower glucose & lipids	7,051	49.2 (36.5–64.1)	55.8% (54.6–56.9)
C1	Higher LDL-C/TC	7,529	53.8 (43.4–63.6)	75.0% (74.1–76.0)
C2	Higher TG, lower HDL-C	6,220	54.5 (44.3–63.6)	47.8% (46.5–49.0)
C3	Marked hyperglycaemia	1,032	60.4 (51.7–67.6)	50.2% (47.1–53.2)

Table S12: (b) Cluster prevalence within sex and age strata under the primary model, and agreement of a K-means model refitted within each stratum (on the frozen projection, canonically relabelled) with the primary labels of that stratum.

Stratum	n	C0	C1	C2	C3	Refit ARI	Refit agreement
women	13,070	30.1%	43.2%	22.7%	4.0%	0.731	90.2%
men	8,762	35.6%	21.4%	37.1%	5.9%	0.565	81.7%
age 18 39	4,771	48.4%	29.4%	20.7%	1.5%	0.635	86.7%
age 40 59	9,427	26.1%	37.2%	32.2%	4.5%	0.856	94.7%
age 60 plus	7,634	29.9%	34.4%	28.8%	7.0%	0.821	93.5%

Table S13. Arithmetic-consistency failures by source export

Table S13: (a) Records failing the arithmetic-consistency screen (TC $<$ LDL-C+HDL-C$-$0.8), counted among records eligible for that screen (after deduplication, the adult filter and the plausibility windows).

Sub-cohort	Eligible records	Failures	Rate
Regional centres (Almaty + Astana)	10,456	0	0.000%
Network extract, identifier-unique	11,416	22	0.193%

Fisher's exact test: $P = 7e - 07$.

Table S13: (b) The same comparison as a function of the tolerance. A physically impossible excess of LDL-C plus HDL-C over total cholesterol begins at zero; the deployed screen rejects only excesses above 0.8 mmol/L. The 0.8 value was chosen ad hoc before this study; the excess is present in every export, so the screen as implemented removes the most extreme physically impossible profiles and no more.

Tolerance (mmol/L)	Almaty	Astana	Network	Fisher P	Retained in cohort
0.0	2.81%	2.7%	4.52%	4.6e-23	866 (3.97%)
0.2	0.35%	0.24%	1.01%	1.0e-18	159 (0.73%)
0.5	0.03%	0.0%	0.24%	1.5e-09	16 (0.07%)
0.8	0.0%	0.0%	0.14%	8.9e-08	0 (0.0%)

Table S14. Per-feature transport statistics

Table S14: Kolmogorov–Smirnov statistics between matched clusters (training versus identifier-disjoint cohort), summarized per analyte from Table S10d.

Analyte	Mean KS	Maximum KS
Glucose	0.1097	0.1483
TC	0.0601	0.0880
LDL-C	0.0585	0.0752
HDL-C	0.0520	0.0602
TG	0.0374	0.0460

Table S15. Flags fired at the cluster median profiles

Table S15: Reference-interval flags (corrected table, Appendix A of the article) evaluated at each cluster median profile, using the median age of that cluster. AC: atherogenic coefficient. The median C2 patient raises no LDL-C or triglyceride flag, which is why interval-by-interval flagging recovers only a quarter of C2 (Table S11c).

Cluster	Age used	HIGH flags	LOW flags
C0	49	—	—
C1	54	TC	—
C2	54	TC, AC	—
C3	60	glucose, TC, AC	—

Table S16. Per-patient assignment margin

Table S16: Relative assignment margin $(d_2 - d_1)/d_1$, where d_1 and d_2 are the distances to the nearest and second-nearest centroid in the PCA space. A margin near zero places the patient on the boundary between two clusters. The border flag of the deployed reports (assignment distance $> 1.5 \times$ the cluster median) marks 16.53% of patients; of the patients with a margin below 0.10, only 34.4% carry the border flag, so the two quantities measure different things (distance from the centre versus closeness to a neighbour) and both are written to the output record.

Cluster	Median	Q1–Q3	<0.10	<0.25	Border flag	Second-nearest cluster
C0	0.77	0.34–1.41	8.3%	19.7%	16.8%	C1 68%, C2 31%
C1	0.70	0.28–1.38	10.0%	22.9%	15.0%	C2 54%, C0 46%
C2	0.58	0.25–1.13	10.3%	25.0%	18.3%	C1 70%, C0 26%
C3	1.03	0.47–1.75	7.6%	16.4%	14.6%	C2 88%, C0 12%
All	0.70	0.29–1.33	9.4%	22.2%	16.53%	

Table S17. Analytes not used in clustering, by cluster

Table S17: Median (IQR) of analytes that were exported with the panel but took no part in clustering, by primary cluster; Kruskal–Wallis P across clusters. These analytes are ordered only with the full and extended panel configurations (availability by product: full 100.0%; full (promotional) 100.0%; extended 100.0%; extended (promotional) 100.0%; standard 0.0%; standard (promotional) 0.0%), so the subset is selected by product choice, not at random. Units as exported by the laboratory information system; the hs-CRP unit is being confirmed with the laboratory.

Analyte	n	C0	C1	C2	C3	KW P
ApoB (g/L)	2,123	0.8 (0.69–0.91)	1.11 (0.975–1.25)	1.25 (1.11–1.43)	1.165 (0.963–1.41)	4.3e-229
ApoA1 (g/L)	2,123	1.37 (1.21–1.53)	1.61 (1.465–1.805)	1.36 (1.22–1.49)	1.44 (1.28–1.585)	1.2e-97
Lp(a)	129	10.895 (4.838–54.275)	17.7 (8.675–51.665)	22.4 (9.63–90.3)	12.5 (5.25–73.345)	2.8e-01
hs-CRP (mg/L)	2,049	0.137 (0.06–0.34)	0.11 (0.057–0.279)	0.22 (0.109–0.45)	0.343 (0.138–0.653)	4.9e-25
Troponin I	336	1.3 (1.3–2.1)	1.3 (1.3–2.25)	1.3 (1.3–2.575)	1.45 (1.3–5.425)	5.2e-01
ApoB/ApoA1 ratio	2,123	0.587 (0.478–0.703)	0.684 (0.58–0.821)	0.929 (0.793–1.096)	0.8 (0.663–0.99)	3.0e-155

Number of measured records per cluster — ApoB (g/L): C0 665, C1 719, C2 647, C3 92; ApoA1 (g/L): C0 665, C1 719, C2 647, C3 92; Lp(a): C0 32, C1 51, C2 39, C3 7; hs-CRP (mg/L): C0 644, C1 694, C2 624, C3 87; Troponin I: C0 102, C1 108, C2 112, C3 14; ApoB/ApoA1 ratio: C0 665, C1 719, C2 647, C3 92. Share with ApoB $\geq$ 1.0 g/L among those measured: C0 1.0%, C1 6.7%, C2 9.3%, C3 6.4%.

Table S18. Collection sites

Table S18: Cluster prevalence and composition by collection site (sites with at least 200 records; 57 sites in total, 3 in the Almaty export, 5 in the Astana export, 49 in the network extract). Between-site heterogeneity of cluster prevalence: χ^2=413.7, 75 d.f., Cramér's V=0.084.

Site	Export	n	Women	Age	C0	C1	C2	C3
70	almaty	4,806	60.7%	51.0	32.0%	37.8%	26.8%	3.4%
10	astana	4,130	57.8%	49.4	32.1%	35.8%	27.9%	4.3%
62	network	1,351	61.3%	58.4	36.6%	29.0%	29.2%	5.2%
61	network	1,157	62.7%	60.6	38.2%	26.0%	30.0%	5.8%
74	almaty	833	64.9%	55.0	35.4%	37.3%	23.2%	4.1%
37	network	721	59.2%	53.5	29.4%	39.7%	26.6%	4.3%
50	network	585	57.8%	54.5	29.7%	36.1%	29.1%	5.1%
24	network	539	61.2%	54.5	23.2%	46.2%	27.8%	2.8%
42	network	514	55.3%	53.1	33.5%	37.9%	24.9%	3.7%
94	network	450	55.8%	51.5	42.4%	23.1%	28.4%	6.0%
19	astana	432	61.3%	50.9	37.0%	32.6%	26.2%	4.2%
47	network	401	59.9%	57.0	23.7%	31.4%	35.9%	9.0%
90	network	348	54.3%	52.6	39.9%	30.5%	23.3%	6.3%
41	network	337	60.2%	56.2	25.2%	33.5%	32.6%	8.6%
52	network	329	61.4%	53.9	30.7%	34.0%	27.7%	7.6%
40	network	286	54.2%	57.5	28.3%	38.5%	28.3%	4.9%
35	network	244	50.4%	52.2	34.8%	29.9%	32.4%	2.9%
63	network	237	61.6%	55.6	24.9%	32.1%	37.6%	5.5%
93	network	227	55.1%	52.0	33.0%	25.1%	35.2%	6.6%
26	network	222	56.8%	53.8	24.8%	41.0%	28.8%	5.4%
48	network	217	59.0%	60.8	33.2%	30.9%	31.8%	4.1%
98	network	214	58.4%	55.5	35.5%	15.4%	36.9%	12.1%
92	network	212	58.0%	53.1	39.6%	21.7%	34.4%	4.2%
28	network	210	69.0%	58.1	24.3%	41.9%	31.0%	2.9%
75	network	205	62.9%	58.2	28.3%	27.3%	38.0%	6.3%
36	network	204	61.3%	51.8	31.9%	37.3%	27.9%	2.9%

Identifiers are laboratory order numbers, not person identifiers. Records that share sex, age to the day and collection site with another record: 1,217 (5.6%, at most 3 per key); this is an upper bound on repeat orders by the same person, since unrelated people can share the key. The promotional variant of the panel accounts for 84.3% of the pooled cohort (84.6% of the centres, 84.1% of the network-unique cohort); cluster prevalence among promotional versus non-promotional records: C0 31.8% vs 34.9%, C1 34.5% vs 34.5%, C2 28.9% vs 26.5%, C3 4.9% vs 4.1%.

Table S19. Reference intervals: corrected table (in use) and deployed table (before this study)

Table S19: Age- and sex-specific reference intervals of the interpretation layer as executed by the code (mmol/L; AC dimensionless), enumerated by probing the reference predicates on an age grid. The corrected table repairs the two glucose defects of the deployed table (upper limit 6.38 for ages 61–70; lower limit rising with age); all other rows are identical. Rows differing between the two tables are marked.

Analyte	Sex, age band	Low	High	Deployed low	Deployed high
glucose	any, 0–13	3.33	5.55	3.33	5.55
glucose	any, 14–60	3.89	5.83	3.89	5.83
glucose	any, 61–100	3.89	6.1 †	—	—
TC	any, 0–0	1.37	3.5	1.37	3.5
TC	any, 1–13	3.11	5.18	3.11	5.18
TC	any, 14–17	3.11	5.44	3.11	5.44
TC	any, 18–100	3.63	5.2	3.63	5.2
TG	any, 0–66	0.0	2.3	0.0	2.3
TG	any, 67–100	0.0	3.7	0.0	3.7
HDL-C	any, 0–19	0.78	1.68	0.78	1.68
HDL-C	M, 20–100	0.78	1.81	0.78	1.81
HDL-C	F, 20–30	0.78	1.94	0.78	1.94
HDL-C	F, 31–40	0.78	2.07	0.78	2.07
HDL-C	F, 41–100	0.78	2.2	0.78	2.2
LDL-C	M, 0–29	1.55	4.53	1.55	4.53
LDL-C	M, 30–39	2.07	4.92	2.07	4.92
LDL-C	M, 40–49	2.33	5.31	2.33	5.31
LDL-C	M, 50–70	2.33	5.57	2.33	5.57
LDL-C	M, 71–100	2.33	4.92	2.33	4.92
LDL-C	F, 0–40	1.81	4.4	1.81	4.4
LDL-C	F, 41–50	2.07	4.92	2.07	4.92
LDL-C	F, 51–60	2.33	5.7	2.33	5.7
LDL-C	F, 61–70	2.59	6.09	2.59	6.09
LDL-C	F, 71–100	2.46	5.57	2.46	5.57
AC	any, 0–19	0.0	3.0	0.0	3.0
AC	M, 20–29	0.0	2.5	0.0	2.5
AC	M, 30–39	0.0	3.0	0.0	3.0
AC	M, 40–59	0.0	3.5	0.0	3.5
AC	M, 60–100	0.0	3.0	0.0	3.0
AC	F, 20–30	0.0	2.2	0.0	2.2
AC	F, 31–100	0.0	3.0	0.0	3.0

† differs from the deployed table, or the deployed table had no identical band at these ages. Pediatric bands are retained for inference completeness; the study cohort is adults only. Under the deployed table the cohort carried 616 LOW glucose flags, of which 422 were at or above 3.89 mmol/L, and 3,852 HIGH flags; under the corrected table 194 LOW (0 non-hypoglycaemic) and 4,120 HIGH. Reassuring C0 passports issued at a glucose of 6.1 mmol/L or above: 28 under the deployed table, 0 under the corrected table (identical under the five-cluster configuration: 28 and 0); at the ADA prediabetes threshold of 5.6 mmol/L 401 such passports remain under the corrected table, because the upper limit for ages 14–60 (5.83 mmol/L) lies above that threshold.

Table S20. Data dictionary

Table S20: Variables of the source exports and of the analysis, with the field names as they appear in the laboratory information system (LIS) exports, units, transformations and valid ranges. The network extract concatenates several LIS schemas, so one analyte can arrive under two field names; synonymous fields are folded into the canonical field, first non-missing value winning.

Variable	LIS field(s)	Unit	Handling / valid range
Laboratory identifier	`internal_nr`	—	Order number; deduplication key; two leading digits encode the collection site. Not a person identifier.
Product code / name	`profile_code`, `profile_name`	—	Panel configuration (standard, full, extended; promotional variant flagged in the name). Descriptive use only.
Sex	`sex`	—	Harmonised from numeric and textual encodings to M/F; used only for reference-interval selection.
Age	`age`	years	Stored in days for most records; values above 200 divided by 365.25. Valid 18–100 for the cohort.
Glucose	`Глюкоза`	mmol/L	Nominally fasting (patients are instructed to attend fasting in the morning; not verified). Plausibility 1.0–40.0. Clustering feature.
Total cholesterol	`Холестерин`; synonyms `Холестерин общий`, `Общий холестерин`	mmol/L	Plausibility 1.0–20.0. Clustering feature.
Triglycerides	`Триглицериды`	mmol/L	Plausibility 0.1–30.0. Clustering feature.
HDL-C	`Холестерин ЛПВП`	mmol/L	Plausibility 0.2–5.0. Clustering feature.
LDL-C	`Холестерин ЛПНП`	mmol/L	Plausibility 0.3–15.0. Not a deterministic function of the other analytes (Table S8). Clustering feature.
Atherogenic coefficient	`Индекс атерогенности`; synonym `Коэффициент атерогенности`	—	(TC − HDL-C)/HDL-C; recomputed where missing. Interpretation layer only.
Apolipoprotein B / A1	`Апо B`, `Апо A1`; synonyms `Аполипопротеин B`, `Аполипопротеин A1`	g/L	Full/extended panel only. Not used in clustering (Table S17).
Lipoprotein(a)	`Липопротеин (а)`	as exported	Full/extended panel only. Not used in clustering.
hs-CRP	`СРБ кардио`; synonym `С-Реактивный белок кардио (высокочувствительный)`	as exported	Full/extended panel only. Not used in clustering.
Troponin I	`Тропонин-I`	as exported	Full/extended panel only. Not used in clustering.
Transformed features	—	—	$\log(1+x)$, then median/IQR scaling fitted on the development cohort, then PCA (3 components, ≥90% variance).
Cluster (K-means)	`cluster_raw`	—	Canonical label C0–C3 after the deterministic relabeling rule.
Delivered category	`cluster_final`	—	After the two-stage reconciliation (Table S11a).
Assignment distance, margin	`dist_centroid`, `dist_second_centroid`, `margin`, `is_border`	PCA units	Border flag: distance > 1.5 × cluster median; margin $(d_2 - d_1)/d_1$ (Table S16).
Report template	`blank_id`, `rules_fired`, `active_flags_clean`, `combos`	—	Cluster passport or single-deviation template F1–F13; rule identifiers fired; flags and combination rules reported alongside the passport.
Collection date, instrument, reagent lot	—	—	Not present in the exports; the study period cannot be stated.

Table S21. Worked patient-level examples

Table S21: Five de-identified records (sex, five-year age band, analyte values) traced through the pipeline: reference-interval flags, K-means cluster, distance to the assigned centroid against the cluster median, border flag, margin and second-nearest cluster, reconciliation rules fired, delivered category and report template. Cases E and F illustrate the glucose decision limit from both sides: with glucose HIGH and at or above 7.0 mmol/L together with high triglycerides the patient is moved to the marked-hyperglycaemia passport (E); with a HIGH glucose flag below 7.0 mmol/L the patient keeps the lipid-pattern passport and the glucose flag is reported alongside it (F).

Field	A	B	C	D	E	F
Sex, age band	M, 75-79	M, 70-74	F, 60-64	F, 60-64	M, 35-39	M, 45-49
Glucose (mmol/L)	4.71	6.95	5.21	6.35	7.06	6.48
TC (mmol/L)	4.69	2.88	6.88	8.37	5.94	7.24
TG (mmol/L)	1.55	0.65	3.31	2.17	3.03	4.5
HDL-C (mmol/L)	1.47	1.01	1.19	1.19	1.22	1.07
LDL-C (mmol/L)	2.5	1.92	4.31	6.34	3.53	4.8
Flags (non-normal)	none	Glucose HIGH, TC LOW, LDL-C LOW	TC HIGH, TG HIGH, AIP HIGH	Glucose HIGH, TC HIGH, LDL-C HIGH, AIP HIGH	Glucose HIGH, TC HIGH, TG HIGH, AIP HIGH	Glucose HIGH, TC HIGH, TG HIGH, AIP HIGH
K-means cluster	C0	C0	C2	C2	C2	C2
Distance / cluster median	0.812 / 1.008	2.109 / 1.008	0.918 / 1.058	1.74 / 1.058	1.452 / 1.058	1.741 / 1.058
Border flag	no	yes	no	yes	no	yes
Margin; second-nearest	0.639; C1	0.587; C2	1.327; C1	0.471; C1	0.866; C3	0.87; C1
Rules fired	none	none	none	B4-06	R4-07	none
Delivered category	C0	C0	C2	C1	C3	C2
Report template	passport C0	F2	passport C2	passport C1	passport C3	passport C2

Table S22. Serialized parameters of the deployed model

Table S22: Every numerical parameter needed to assign a new five-analyte profile to a cluster without the source code. Transform: $x_j = \log(1 + v_j)$ for the five analytes in the order glucose, TC, TG, HDL-C, LDL-C; $z_j = (x_j - c_j)/s_j$ with the robust-scaler centre c and scale s; $y = W(z - \mu)$ with the PCA components W (rows) and mean μ; the cluster is the nearest centroid in y-space (raw K-means order, mapped to the canonical label by the relabeling map); the border flag compares the distance to the assigned centroid with 1.5× the cluster median assignment distance.

Parameter	Value
Scaler centre c (log scale)	1.829376, 1.838961, 0.819780, 0.837247, 1.467874
Scaler scale s (log scale)	0.140134, 0.269664, 0.386081, 0.223144, 0.338837
PCA mean μ	0.228064, -0.034696, 0.144321, 0.053558, -0.055080
PCA component 1 (row of W)	0.903936, 0.095745, 0.358849, -0.201197, 0.066935
PCA component 2 (row of W)	-0.164624, 0.682108, 0.207049, 0.173592, 0.659261
PCA component 3 (row of W)	-0.390633, -0.070107, 0.656708, -0.638144, -0.063225
K-means centre, raw index 0 (y-space)	0.626855, 0.593870, 0.639579
K-means centre, raw index 1 (y-space)	-0.504932, -1.091638, 0.073862
K-means centre, raw index 2 (y-space)	-0.629064, 0.624238, -0.491726
K-means centre, raw index 3 (y-space)	4.260476, -0.672309, -0.783048
Relabeling map raw → canonical	3 → C3, 1 → C0, 2 → C1, 0 → C2
Median assignment distance per canonical cluster	C2: 1.0576, C0: 1.0083, C1: 0.9280, C3: 2.0110
Centroid C0, back-projected (mmol/L)	Glucose 5.163, TC 4.025, TG 1.090, HDL-C 1.268, LDL-C 2.295
Centroid C1, back-projected (mmol/L)	Glucose 5.014, TC 5.941, TG 1.041, HDL-C 1.642, LDL-C 3.879
Centroid C2, back-projected (mmol/L)	Glucose 5.633, TC 5.979, TG 2.228, HDL-C 1.123, LDL-C 3.867
Centroid C3, back-projected (mmol/L)	Glucose 10.698, TC 5.239, TG 2.365, HDL-C 1.103, LDL-C 3.106
Software	Python 3.11, scikit-learn 1.8.0; seed 42; corrected reference table; glucose decision limit 7.0 mmol/L

S23. Pseudocode of the interpretation layer

The following pseudocode, together with the decision table (Table S11a), the reference intervals (Table S19), the templates and combination rules (Appendices B and C of the article) and the serialized transform parameters (scaler centre and scale, PCA components and mean, centroids; `centroids.json` in the deposit), specifies the layer completely, so that every delivered report can be reproduced from the five analyte values, sex and age without access to the source code.

```
INPUT glucose, TC, TG, HDL, LDL (mmol/L); sex in {M, F}; age (years)
CONST BORDER_K = 1.5; GLUCOSE_C3_LIMIT = 7.0; MAX_ITER = 3
      median_dist[c] -- median assignment distance of cluster c (from training)
      RULES_BORDER, RULES_FLAG -- ordered rule lists of Table S11a

1 x <- PCA.transform( RobustScaler.transform( log(1 + [glucose, TC, TG, HDL, LDL]) ) )
2 d <- Euclidean distances from x to the four centroids
3 c <- canonical label of argmin d; d1 <- min d; d2 <- second-smallest d
4 margin <- (d2 - d1) / d1; border <- (d1 > BORDER_K * median_dist[c])
5 AC <- recorded atherogenic coefficient, else (TC - HDL) / HDL
6 for each analyte a in {glucose, TC, TG, HDL, LDL, AC}:
      flag[a] <- HIGH if value > upper(a, sex, age); LOW if value < lower(a, sex, age); else NORMAL
7 glu_hi <- flag[glucose] = HIGH; glu_c3 <- glu_hi and glucose >= GLUCOSE_C3_LIMIT
  tg_hi, tc_hi, ldl_hi <- flag = HIGH; hdl_lo <- flag[HDL] = LOW; n_flags <- count(flag != NORMAL)
8 if border: -- stage 1, border cases only
      for rule in RULES_BORDER with rule.source = c:
          if rule.condition(flags): c <- rule.target; break
9 repeat at most MAX_ITER times: -- stage 2, everyone, to a fixed point
      c_new <- c
      for rule in RULES_FLAG with rule.source = c:
          if rule.condition(flags): c_new <- rule.target; break
      if c_new = c: break; c <- c_new
10 conflict <- (TC < LDL + HDL - 0.8) or |AC - (TC - HDL)/HDL| > 2.0
11 sig <- flags != NORMAL; drop TC flag if concordant with LDL flag; drop AC flag if discordant with HDL flag
```

```
12 template <- F7 if conflict
            else passport(c) if c in {C2, C3}
            else passport(C0) if sig empty
            else single-deviation template of the only flag if |sig| = 1
            else passport(C1) if c = C1
            else single-deviation template of the highest-priority flag (glucose > TG > LDL > HDL > TC > AC)
13 combos <- all satisfied combination rules COM-1..COM-8 (narrative fragments only)
14 OUTPUT c (delivered category), template, sig (flags reported alongside), combos,
        d1, d2, margin, border, rules fired
```

STROBE checklist (cross-sectional studies)

Item	Where addressed
1 Title and abstract	Title (descriptive phenotyping; no risk-stratification claim); structured abstract stating the retrospective design, the reporting-granularity decision and the absence of clinical endpoints
2 Background/rationale	Introduction, paragraphs 1–3
3 Objectives	Introduction, final paragraph
4 Study design	Methods 2.1 (retrospective, non-interventional analysis of routinely collected results); Limitations
5 Setting	Methods 2.1 (laboratory network: 22 laboratories in 20 regions, more than 800 collection points; collection dates not exported)
6 Participants / eligibility	Methods 2.1–2.2; Figure 1; Table S1
7 Variables	Methods 2.1, 2.3; Table S20 (data dictionary); Appendix A
8 Data sources / measurement	Methods 2.1 (LIS exports; analyser platform; LDL-C provenance, Table S8); Appendix A
9 Bias	Methods 2.2 (quality filters); Limitations (selection, analytical heterogeneity, fasting, therapy)
10 Study size	Results 3.1; Figure 1
11 Quantitative variables	Methods 2.3 (log transform, robust scaling); Sensitivity (Table 5, Table S9)
12(a) Statistical methods	Methods 2.4–2.7
12(b) Subgroups/interactions	Sex and age strata: Results 3.4, Table S12
12(c) Missing data	Methods 2.2 (complete-case; no imputation); Table S1
12(d) Sampling strategy	Methods 2.1 and Limitations (self-selected purchasers of a promotional panel; exports not a random sample of the network)
13(a) Numbers at each stage	Results 3.1; Figure 1; Table S1
13(b) Reasons for exclusion	Figure 1; Table S1
13(c) Flow diagram	Figure 1
14(a) Descriptive data	Table 1; Table 3; Table S12
14(b) Missing data per variable	Table S1
15 Outcome data	Not applicable (no outcome variable; stated in the abstract and Limitations)
16 Main results	Results 3.2–3.8; Tables 2–5; absolute differences with 95% intervals (Table 4, Table S10)
17 Other analyses	Sensitivity (3.7), strata (Table S12), collection sites (Table S18), analytes not used in clustering (Table S17)
18 Key results	Discussion 4.1
19 Limitations	Discussion, Limitations
20 Interpretation	Discussion 4.2–4.8
21 Generalisability	Discussion, Population and selection; Limitations
22 Funding	Funding statement
